# Serum metabolomics analysis reveals a novel association between maternal metabolism and fetal survival in sows fed diets containing differing methionine levels and sources

**DOI:** 10.1016/j.aninu.2024.07.008

**Published:** 2024-10-26

**Authors:** Rui Zhou, Li Zhe, Yves Mercier, Liang Hu, Ran Li, Hong Chen, Xiaoling Zhang, Lingjie Huang, Lun Hua, Yong Zhuo, Jian Li, Shengyu Xu, Yan Lin, Bin Feng, Lianqiang Che, De Wu, Zhengfeng Fang

**Affiliations:** a Key Laboratory for Animal Disease-Resistance Nutrition of China Ministry of Education, Animal Nutrition Institute, Sichuan Agricultural University, Chengdu 611130, China; b Adisseo France S.A.S., Commentry F-03600, France; c Key Laboratory of Agricultural Product Processing and Nutrition Health (Co-construction by Ministry and Province), Ministry of Agriculture and Rural Affairs, College of Food Science, Sichuan Agricultural University, Ya'an 625014, China

**Keywords:** Amino acid metabolism, Methionine, Gestation sow, Glycolipid metabolism, Oxidative stress, Reproductive performance

## Abstract

Methionine (Met) metabolism is vital for one carbon metabolism, redox status and fetal development. Hence, this study investigated the effects of different levels and sources of Met on maternal metabolism, anti-oxidative capacity and fetal survival in pregnant sows. Forty primiparous sows were assigned to the following four groups: control group (basal diet, CON), 1.5S-OHMet group (supplemented methionine hydroxy analogue [OHMet] at 1.5 g/kg diet), 3.0S-OHMet group (supplemented OHMet at 3.0 g/kg diet), and 3.0S-Met group (supplemented L-Met at 3.0 g/kg diet) (*n* = 10). The trial lasted from day 60 of gestation to the farrowing day. Maternal 1.5S-OHMet consumption had the lowest stillborn ratio and the highest serum glucose levels during farrowing. Further analysis revealed that dietary 1.5S-OHMet consumption elevated the serum contents of glucose-6-phosphate, citric acid, butyric acid, malic acid, 3-methyladenine, 1-methyladenosine, ferulic acid and salicylic acid, but reduced the serum contents of succinic acid, oxoglutaric acid, 9(S)-hydroperoxylinoleic acid, 13(S)-hydroperoxy-octadecatrienoic acid, uric acid and urea nitrogen when compared to contents observed in the 3.0S-OHMet and 3.0S-Met groups (*P* < 0.05). Serum metabolomics analysis was conducted to determine the enriched differential metabolites and an enrichment analysis was performed using Kyoto Encyclopedia of Genes and Genomes pathway enrichment analysis. The results showed that the enriched metabolites were mainly associated with central carbon metabolism, amino acid metabolism, lipid metabolism, and nucleotide metabolism. Moreover, maternal 3.0S-OHMet or 3.0S-Met consumption upregulated the trans-methylation pathway by elevating the S-adenosyl-methionine (SAM) level and the ratio of SAM to S-adenosyl-homocysteine (*P* < 0.05) at day 114 of gestation, while increasing homocysteine concentration (*P* < 0.001). However, compared to the 3.0S-Met group, maternal 3.0S-OHMet consumption elevated fetal survival and glutathione peroxidase (*P* < 0.05). Thus, this study provided new insights into the mechanisms through which sows fed with a 1.5S-OHMet diet during mid-to late-gestation period had high fetal survival, such as improvements in maternal amino acid, nucleotide and glycolipid metabolism.

## 1 Introduction

Methionine (Met), an essential amino acid, plays a pivotal role in the nutrition and health of gestational animals (Fang et al., 2010a; Korsmo and Jiang, 2021), as it is involved in the one-carbon transfer reaction, DNA and RNA methylation, and anti-oxidative activity (Kalhan and Marczewski, 2012). Therefore, given that the fetus largely depends on its mother for the necessary nutrients to sustain rapid cell division during the early stages of its life, ensuring a balanced intake of amino acids in the mother's diet is a crucial long-term objective (Rees et al., 2006a; Zhou et al., 2024a). Recent studies have highlighted the importance of an adequate Met intake for improving fetal survival rates and reducing the risk of intrauterine growth restriction (Bin et al., 2018; Xia et al., 2019; Zhou et al., 2024b). However, excessive dietary Met is associated with increased fetal mortality due to elevated levels of homocysteine (Hcy), a byproduct of Met metabolism (Obeid and Herrmann, 2009; Yang et al., 2019). In addition, researchers have observed a substantial increase in Hcy concentration in sows during late gestation (Xia et al., 2021).

2-Hydroxy-4-methylthiobutanoic acid (OH-Met), as one possible source of Met, shows higher utilization in parenteral tissues, and can be easily metabolized via the trans-sulphuration pathway. OH-Met also leads to lower Hcy accumulation when compared to DL-Met (Xie et al., 2007; Fang et al., 2010b; Li et al., 2014). Our previous study reported that a lower amount of OH-Met undergoes first-pass metabolism in the intestine than an equivalent mole of Met (Fang et al., 2010b,c). Hence, OH-Met is hypothesized to be more effective in mitigating oxidative stress-induced damage than DL-Met. However, it remains unclear whether different Met sources induce similar dose-dependent effects on Hcy production in pregnant animals. This knowledge gap prompted us to conduct a comprehensive investigation to reveal the mechanisms that underlie the beneficial effects of optimal Met supplementation on maternal and fetal health. A previous study demonstrated that maternal Met supplementation altered the levels of certain metabolic products associated with the health of pregnant sows (Bin et al., 2018). However, the specific mechanisms underlying this phenomenon remain largely unknown. Therefore, further research is warranted to elucidate the mechanisms through which Met supplementation improves reproductive outcomes in sows.

The present study aimed to reveal whether increasing the consumption of OH-Met or L-Met can improve maternal metabolism. For this purpose, we analyzed serum metabolites through an untargeted metabolomics approach, with particular emphasis on Met metabolites, glycolipid and amino acid metabolism, and the antioxidative capacity of sows. The findings of this study provided novel insights into changes in maternal health in response to maternal Met supplementation.

## 2 Materials and methods

### 2.1 Animal ethics statement

The experimental protocol was approved by the Institutional Animal Care and Use Committee of Sichuan Agricultural University (Ya'an China) (approval no. SYXK-Chuan-2014 to 184).

### 2.2 Animals, experimental design and diets

Forty primiparous sows [Duroc × (Landrace × Yorkshire)] with similar body weight (BW) (154.46 ± 1.60 kg) were selected and fed the same control (CON) diet from day 1 of gestation (G1) to G59. The CON diet was formulated according to the nutrition requirements of gestating sows as recommended by the National Research Council (NRC, 2012). From G60 to G114, sows were randomly assigned to one of four dietary treatments (*n* = 10): CON group, basal diet (Table 1); 1.5S-OHMet group, basal diet + 1.5 g/kg OHMet; 3.0S-OHMet group, basal diet + 3.0 g/kg OHMet; and 3.0S-Met group, basal diet + 3.0 g/kg L-Met. The OH-Met was provided by Adisseo (purity 88%, Rhodimet AT88, Adisseo Life Science, Shanghai, China) and the L-Met (purity 99%) was obtained from Cheil Jedang Biochemical Co., Ltd (Shenyang, China). The Met levels in the 1.5S-OHMet, 3.0S-OHMet and 3.0S-Met groups were based on a previous study (Bin et al., 2018).

**Table 1:** Ingredients and nutrient compositions of the diets of sows (as-fed basis, g/kg).

Ingredients	Phase 1	Phase 2	Nutrient levels^2^	Phase 1	Phase 2
	G60-G90	G91-G114		G60-G90	G91-G114
Corn	689.11	642.58	Gross energy, MJ/kg	16.51	16.52
Wheat bran	70.00	55.00	Dry matter	877	870
Rice bran meal	60.00	60.00	Crude protein	102	128
Soybean meal	41.50	97.00	Crude fiber	52.9	46.3
Bean curd	69.30	50.90	Ether extract	40.6	52.5
Fish meal		20.00	Ash	42.1	48.1
Soybean oil	40.00	40.00	Lys	6.3	8.1
L-Lys-HCl (98.5%)	2.602	2.324	Met	1.3	2.0
L-Met (99%)	0.112	0.469	Met + Cys	2.3	3.2
L-Thr (98.5%)	0.892	0.966	Trp	0.9	1.2
Trp	0.084	0.15	Ile	3.3	4.3
Dicalcium phosphate	8.041	10.528	Val	4.4	5.7
Limestone	8.733	10.461	Thr	4.4	5.6
Sodium chloride	4.00	4.00	Calcium	6.1	8.3
Choline chloride (50%)	1.70	1.70	Total phosphorus	4.9	6.2
Premix^1^	3.925	3.925			

G60 = day 60 of gestation; G90 = day 90 of gestation; G91 = day 91 of gestation; G114 = day 114 of gestation.

1 Supplied the following per kilogram premix: VA 6000 IU; VD_3_ 1200 IU; VE 50 IU; VB_1_ 1 mg; VB_2_ 3.6 mg; VB_6_ 1.8 mg; VB_12_12.5 μg; niacin 20 mg; folate 2 mg; iron 316 mg; copper 183 mg; zinc 289 mg; manganese 169 mg; iodine 0.3 mg.

2 Analyzed values.

The present study was divided into two phases: phase 1 (G60 to G90) and phase 2 (G91 to G114). Sows were individually housed in a 2.6 m × 0.6 m pen from G60 to G107 and then transferred to adjustable farrowing cages of size 2.2 m × 1.5 m. Starting from G60, all sows systematically received one of the four diets twice daily (at 08:00 and 16:00, half of the daily meal each time, total 2.6 kg), along with free access to water. Because of dystocia, two sows were removed from the 3.0S-OHMet and 3.0S-Met groups.

### 2.3 Sample collection and analysis

The total number of piglets born, number of piglets born alive, individual birth weight of piglets, and duration of farrowing were immediately recorded after delivery. The fasting blood samples of sows were collected via the ear vein on G90 and at farrowing. The umbilical cord blood samples were collected from the placentas of piglets with a BW closest to the litter's average BW. All the blood samples were centrifuged at 3000 × *g* for 15 min, and the serum was separated and stored at −20 °C until analysis, or at −80 °C for metabolic analysis.

The diets in this study were evaluated for nutritional components, including dry matter (method 930.15; AOAC, 2006), crude protein (method 984.13; AOAC, 2006), crude fiber (method 978.10; AOAC, 2006), ash (method 942.05; AOAC, 2006), gross energy (GE), amino acid, calcium (method 968.08; AOAC, 2006) and total phosphorus content (method 946.06; AOAC, 2006). GE was measured using an adiabatic oxygen bomb calorimeter (Parr Instruments Co., Moline, IL, USA). For amino acid analysis, most amino acid were hydrolyzed in 6 mol/L HCl at 110 °C for 24 h and analyzed with an Amino Acid Analyzer (Hitachi L-8900, Hitachi Ltd., Tokyo, Japan). Met and Cys were oxidized with cold performic acid, then hydrolyzed in 7.5 mol/L HCl at 110 °C for 24 h before analysis with the same Amino Acid Analyzer. Tryptophan was determined by HPLC (Agilent 1200 Series, Agilent Technologies Inc., Santa Clara, CA, USA) after LiOH hydrolysis at 110 °C for 22 h.

### 2.4 Serum biochemical indicators analysis

The following biochemical indicators were measured using commercially available kits (Shanghai Kehua Bioengineering Co., Ltd., China): homocysteine (Hcy), total bile acid (TBA), total cholesterol (TC), triglyceride (TG), glucose (GLU), non-esterified fatty acid (NEFA), low density lipoprotein cholesterol (LDL-C), and high-density lipoprotein cholesterol (HDL-C). The analyses of these indicators were conducted using automatic biochemical analyzer (Hitachi 3100, Hitachi High-Technologies Corporation, Tokyo, Japan).

### 2.5 Serum amino acid analysis

For free amino acid analysis, the frozen serum was thawed at 4 °C. The serum sample (1 mL) and 2.5 mL of 7.5% (w/v) trichloroacetic acid solution were mixed thoroughly and centrifuged at 12,000 × *g* at 4 °C for 15 min. Subsequently, the supernatant was collected for amino acid analysis by ion-exchange chromatography using an automatic amino acid analyzer (Hitachi L-8800, Hitachi High-Technologies Corp., Japan).

### 2.6 S-Adenosyl methionine (SAM) and S-adenosyl homocysteine (SAH) analysis

The serum sample (400 μL) and 40 μL trichloroacetic acid solution (400 g/L) were mixed and centrifuged at 14,000 × *g* for 20 min at 4 °C, and the supernatant was collected. SAM (#A4377) and SAH (#A9384) standards were purchased from Sigma–Aldrich (Germany) (Zhong et al., 2021). The mobile phase (pH 3.1) comprised 40 mmol/L NaH_2_PO_4_, 8 mmol/L sodium heptane sulfonate, and 18% (v/v) methanol. The SAM and SAH contents in serum samples were determined using ultra-performance liquid chromatography (UPLC) system equipped with a Waters ACQUITY UPLC BEH C18 column (150 mm × 2.1 mm, 1.7 μm).

### 2.7 Antioxidant capacity analysis

Commercial test kits (NJJBIO, Nanjing, China) were employed to assess the levels of glutathione peroxidase (GSH-Px, #A005-1-2), total superoxide dismutase (T-SOD, #A001-3-2), malondialdehyde (MDA, #A003-1-12), and catalase (CAT, #A007-1-1) in serum samples. The methods used are described in our previous study (Zhong et al., 2021).

### 2.8 Untargeted metabolomics analysis

The vortexed serum samples were accurately transferred into centrifuge tubes, and 400 μL methanol (−20 °C) was added, followed by vortexing for 1 min. After centrifugation, the supernatant was transferred to new centrifuge tubes, concentrated, and dried (4 °C). Finally, the samples were dissolved in 150 μL of a 2-chloro-L-phenylalanine (4 mg/L) solution prepared in 80% (v/v) methanol/water (4 °C), filtered, and transferred into vials for LC-MS detection. The detection methods used are reported in our previous study (Zhong et al., 2021).

### 2.9 Statistical analysis

The data of the survival rate of piglets, stillbirth rate of piglets, and percentage of birth weight were analyzed using the Chi-square test. The remaining data were analyzed using the PROC MIXED procedure in SAS 9.4 software (SAS Institute Inc., Cary, NC, USA). The following model:

*Y*_*ij*_ = *μ*+ *α*_*i*_ + *e*_*ij*__*.*_

In the above models, observed traits were denoted by *Y_ij_*, where *μ* represents the population mean, *α*_*i*_ signified the fixed effect of treatment (*i* = CON, 1.5S-OHMet or 3.0S-OHMet), *e*_*ij*_ denoted the residual. The analysis of treatment levels of linear and quadratic effects was performed using polynomial orthogonal contrasts by the command CONTRAST in SAS. The different dietary Met sources were analyzed by *t*-test. A Pearson's correlation analysis was performed to determine the association of Met metabolism with amino acid concentration and glycolipid metabolism. Data were presented as mean values with standard error of the mean (SEM). The differences were considered significant at *P* < 0.05, and were considered a tendency toward difference at 0.05≤ *P* < 0.10.

## 3 Results

### 3.1 Met supplementation improved reproductive performance

The results showed that no significant differences in the total number of piglets born, number of piglets born alive, average birth weight of all piglets and average pig birth weight (*P* > 0.05, Table 2). The sows fed 1.5S-OHMet had the lowest stillborn rate compared with the other groups (Table 3); moreover, the 1.5S-OHMet group showed an increased survival rate of piglets compared with the 3.0S-Met group (*P* = 0.005, Table 3). In addition, compared with the 3.0S-Met group, sows supplemented with 3.0S-OHMet had a decreased stillborn rate and a lower percentage of piglets with a born weight <1.0 kg (*P* < 0.05, Table 3).

**Table 2:** Effect of increased consumption of methionine on reproductive performance of sows^1^.

Item	CON	1.5S-OHMet	3.0S-OHMet	3.0S-Met	SEM	*P*-value			
						ANOVA	Linear^2^	Quadratic^2^	Met source^3^
BW of sow at G1, kg	156.00	155.80	152.67	153.06	3.385	0.846	0.472	0.709	0.948
Feed intake, kg/d	2.60	2.60	2.60	2.60	0.000	1.000	1.000	1.000	1.000
Total number of piglets born	11.50	11.40	12.89	12.89	0.856	0.581	0.258	0.444	1.000
Number of piglets born alive	9.80	10.50	11.56	10.22	0.938	0.339	0.163	0.866	0.462
Average BW of all piglets, kg	1.68	1.55	1.61	1.49	0.088	0.445	0.596	0.349	0.327
Average BW of alive piglets, kg	1.69	1.56	1.63	1.48	0.093	0.375	0.588	0.317	0.287
Duration of farrowing, min	173.88	151.00	148.50	160.60	20.730	0.970	0.860	0.890	0.189

BW = body weight; G1 = day 1 of gestation.

1 CON = basal diet; 1.5S-OHMet = basal diet + 1.5 g/kg OHMet; 3.0S-OHMet = basal diet + 3.0 g/kg OHMet; 3.0S-Met = basal diet + 3.0 g/kg Met. Each treatment contained 9 to 10 replicates (*n* = 9–10). Data were presented as means with standard error of mean (SEM).

2 3.0S-Met group is excluded from regression analyses.

3 Significant differences (*P* < 0.05) between different Met sources (3.0S-OHMet vs. 3.0S-Met).

**Table 3:** Effect of dietary methionine supplementation on stillbirth rate and birth weight of piglet^1^.

Item	CON	1.5S-OHMet	3.0S-OHMet	3.0S-Met
Survival rate, %	85.22^ab^	92.11^a^	89.66^a^	79.31^b^
Stillborn rate^2^, %	10.09^a^	0.94^c^	6.30^b^	15.60^a^
Birth weight distribution of piglets^2^, %				
<1.0 kg	7.14^b^	13.33^ab^	5.77^b^	16.30^a^
1.0–1.5 kg	35.71	37.14	37.50	34.78
>1.5 kg	57.14^a^	49.52^ab^	56.73^a^	48.91^b^

^a-c^ Within a row, means without a common superscript differ significantly (*P* < 0.05).

1 CON = basal diet; 1.5S-OHMet = basal diet + 1.5 g/kg OHMet; 3.0S-OHMet = basal diet + 3.0 g/kg OHMet; 3.0S-Met = basal diet + 3.0 g/kg Met. Each treatment contained 9 to 10 replicates (*n* = 9–10).

2 The stillbirth rate and birth weight distribution of piglets were analyzed using Chi-square.

### 3.2 Met supplementation changed methionine metabolism

As shown in Table 4, serum Met, Hcy and taurine concentrations increased linearly (*P* < 0.05) following increased consumption of dietary OHMet at G90. The 3.0S-Met group exhibited the lowest ratio of SAM to SAH and the highest SAH concentration compared with the other groups at G90 (*P* < 0.05).

**Table 4:** Effect of increased consumption of methionine on methionine metabolism in serum^1^.

Item	CON	1.5S-OHMet	3.0S-OHMet	3.0S-Met	SEM	*P*-value			
						ANOVA	Linear^2^	Quadratic^2^	Met source^3^
**G90, μmol/L**									
Methionine	27.68^b^	32.78^b^	44.87^a^	44.00^a^	2.896	<0.001	<0.001	0.220	0.884
SAM	0.45	0.46	0.45	0.44	0.008	0.470	0.628	0.315	0.436
SAH	0.63^b^	0.67^b^	0.65^b^	0.78^a^	0.031	0.002	0.533	0.228	0.015
SAM/SAH	0.61^a^	0.54^a^	0.56^a^	0.40^b^	0.033	<0.001	0.247	0.265	0.001
Hcy	19.01^b^	20.01^b^	28.06^a^	24.92^a^	1.100	<0.001	<0.001	0.023	0.091
Cysteine	1.28^b^	1.65^ab^	3.74^a^	2.46^ab^	0.760	0.010	0.030	0.380	0.360
Cystathionine	3.71	3.14	3.98	2.70	0.620	0.440	0.150	0.610	0.070
Taurine	82.4^b^	100.8^ab^	124.8^a^	132.1^a^	11.44	<0.001	0.007	0.540	0.680
Serine	134.90^a^	131.70^ab^	114.66^bc^	105.53^c^	6.413	0.007	<0.001	0.089	0.870
Glycine	2488.2^a^	2435.4^a^	1643.2^b^	1641.7^b^	146.02	<0.001	0.001	0.078	0.910
**G114, μmol/L**									
Methionine	45.74	41.58	52.16	55.50	4.896	0.138	0.250	0.510	0.590
SAM	1.41^b^	1.22^b^	1.75^a^	1.67^a^	0.068	<0.001	0.003	<0.001	0.315
SAH	1.09^a^	1.15^a^	0.89^b^	0.89^b^	0.047	<0.001	0.017	0.025	0.869
SAM/SAH	1.36^b^	1.08^b^	1.97^a^	1.90^a^	0.107	<0.001	0.001	<0.001	0.531
Hcy	36.43^bc^	32.75^c^	43.38^ab^	49.95^a^	2.435	<0.001	0.066	0.025	0.041
Cysteine	14.83^bc^	8.82^c^	20.96^b^	39.08^a^	3.054	<0.001	<0.001	<0.001	<0.001
Cystathionine	13.23^ab^	10.47^b^	14.78^a^	15.78^a^	0.920	0.001	0.080	<0.001	0.630
Taurine	244.37^a^	225.73^a^	199.91^b^	155.02^c^	13.395	<0.001	0.050	0.840	<0.001
Serine	153.71	129.73	156.32	147.42	9.939	0.227	0.546	0.057	0.919
Glycine	869.06^a^	894.58^a^	839.52^a^	690.60^b^	47.789	0.017	0.435	0.293	0.031

G90 = day 90 of gestation; G114 = day 114 of gestation; SAM = S-adenosyl-methionine; SAH = S-adenosyl-homocysteine; Hcy = homocysteine.

^a-c^ Within a row, means without a common superscript differ significantly (*P* < 0.05).

1 CON = basal diet; 1.5S-OHMet = basal diet + 1.5 g/kg OHMet; 3.0S-OHMet = basal diet + 3.0 g/kg OHMet; 3.0S-Met = basal diet + 3.0 g/kg Met. Each treatment contained 9 to 10 replicates (*n* = 9–10). Data were presented as means with standard error of mean (SEM).

2 3.0S-Met group is excluded from regression analyses.

3 Significant differences (*P* < 0.05) between different Met sources (3.0S-OHMet vs. 3.0S-Met).

At G114, compared to the CON and 1.5S-OHMet groups, the 3.0S-OHMet and 3.0S-Met groups exhibited a significant increase in the serum SAM concentration and the SAM to SAH ratio, and a significant reduction in the SAH concentration (*P* < 0.05). The Hcy and cysteine contents increased significantly in the 3.0S-Met group compared to those in the 1.5S-OHMet groups (*P* < 0.05). The Hcy content increased gradually (*P* < 0.001) with prolonged gestation (Fig. S1A). The maternal Hcy level was negatively correlated with the average weight of piglets born alive (*P* = 0.011, *R*^2^ = 0.181, Fig. S1B) and total survival rate of piglets (*P* = 0.048, *R*^2^ = 0.116, Fig. S1C). At G114, the 3.0S-OHMet group showed a higher taurine concentration, but lower Hcy and cysteine concentrations when compared to the 3.0S-Met group (*P* < 0.05).

### 3.3 Met supplementation improved glycolipid metabolism and serum urea nitrogen level

As shown in Table 5, the consumption of 3.0S-OHMet and 3.0S-Met diet significantly increased TG and LDL-C concentrations (*P* < 0.05) at G90, elevated TG and NEFA concentrations (*P* < 0.05) at G114, and increased the TG concentration in the umbilical cord blood as compared to the consumption of the CON diet and the 1.5S-OHMet-rich diet (*P* = 0.001). TBA concentration in umbilical cord blood was increased (*P* = 0.027) in the 3.0S-Met group compared with that in the 1.5S-OHMet group. Moreover, a positive correlation was observed between the maternal Hcy and TBA contents at G114 (*P* = 0.010, *R*^2^ = 0.176, Fig. S1D). As shown in Table 5, maternal GLU concentration at G90 increased linearly and quadratically with increase in OH-Met consumption (*P* < 0.05). In addition, at G114, the GLU concentration was significantly increased in the 1.5S-OHMet group as compared to that in the other groups (*P* = 0.003). The serum urea nitrogen level increased quadratically (*P* = 0.032, Table 5) following the increased consumption of dietary OH-Met. Sows in the 1.5S-OHMet group showed reduced urea nitrogen concentrations as compared to those in the 3.0S-OHMet and 3.0S-Met groups (*P* = 0.025, Table 5).

**Table 5:** Effect of increased consumption of methionine on glycolipid metabolism and serum urea nitrogen levels of sows and umbilical cord blood^1^.

Item	CON	1.5S-OHMet	3.0S-OHMet	3.0S-Met	SEM	*P*-value			
						ANOVA	Linear^2^	Quadratic^2^	Met source^3^
**G90**									
SUN, μmol/L	1657	1624	2441	2019	240.8	0.074	0.090	0.255	0.594
TC, mmol/L	1.92^bc^	1.69^c^	2.69^a^	2.28^ab^	0.158	<0.001	0.002	0.003	0.102
TG, mmol/L	0.55^b^	0.47^b^	0.70^a^	0.74^a^	0.030	<0.001	<0.001	<0.001	0.700
NEFA, mmol/L	0.14^a^	0.11^ab^	0.08^bc^	0.04^c^	0.017	<0.001	0.021	0.553	0.021
LDL-C, mmol/L	0.83^b^	0.64^c^	1.24^a^	1.04^a^	0.053	<0.001	<0.001	0.007	0.074
HDL-C, mmol/L	0.68^a^	0.54^b^	0.79^a^	0.76^a^	0.050	0.004	0.146	0.003	0.757
GLU, mmol/L	3.54^bc^	3.18^c^	4.18^a^	3.94^ab^	0.172	0.001	0.024	0.006	0.380
TBA, μmol/L	10.09	13.01	15.06	12.65	1.606	0.204	0.101	0.352	0.649
**G114**									
SUN, μmol/L	3970^ab^	2319^b^	4915^a^	4775^a^	651.4	0.025	0.670	0.032	0.757
TC, mmol/L	1.99	1.82	1.85	1.70	0.129	0.480	0.684	0.416	0.285
TG, mmol/L	0.48^c^	0.45^c^	0.65^b^	0.79^a^	0.037	<0.001	0.001	0.006	0.039
NEFA, mmol/L	0.86^bc^	0.60^c^	1.32^b^	1.78^a^	0.166	<0.001	<0.001	<0.001	0.241
LDL-C, mmol/L	0.95	0.82	0.80	0.73	0.063	0.157	0.200	0.419	0.278
HDL-C, mmol/L	0.49	0.52	0.47	0.48	0.036	0.687	0.934	0.474	0.864
GLU, mmol/L	2.93^b^	4.14^a^	2.36^b^	2.33^b^	0.369	0.003	0.511	0.008	0.836
TBA, μmol/L	7.17	9.81	14.81	18.27	3.639	0.162	0.104	0.894	0.292
**Umbilical cord serum**									
SUN, μmol/L	4432	3596	4510	5145	691.2	0.348	0.942	0.354	0.093
TC, mmol/L	0.66^b^	0.90^ab^	1.10^ab^	1.11^a^	0.153	0.149	0.043	0.931	0.939
TG, mmol/L	0.39^b^	0.42^b^	0.54^a^	0.65^a^	0.034	0.001	0.002	0.343	0.331
NEFA, mmol/L	0.05	0.04	0.06	0.07	0.013	0.278	0.881	0.351	0.476
LDL-C, mmol/L	0.41	0.42	0.52	0.57	0.064	0.255	0.202	0.576	0.622
HDL-C, mmol/L	0.23	0.34	0.39	0.34	0.050	0.196	0.025	0.556	0.555
GLU, mmol/L	1.15	0.67	0.84	1.07	0.438	0.135	0.909	0.059	0.388
TBA, μmol/L	4.38^ab^	2.83^b^	5.22^ab^	7.74^a^	1.162	0.027	0.550	0.128	0.320

G90 = day 90 of gestation; G114 = day 114 of gestation; SUN = serum urea nitrogen; GLU = glucose; TG = triglyceride; NEFA = non-esterified fatty acids; TC = total cholesterol; LDL-C = low density lipoprotein cholesterol; HDL-C = high density lipoprotein cholesterol; TBA = total bile acid.

^a-c^ Within a row, means without a common superscript differ significantly (*P* < 0.05).

1 CON = basal diet; 1.5S-OHMet = basal diet + 1.5 g/kg OHMet; 3.0S-OHMet = basal diet + 3.0 g/kg OHMet; 3.0S-Met = basal diet + 3.0 g/kg Met. Each treatment contained 9 to 10 replicates (*n* = 9-10). Data were presented as means with standard error of mean (SEM).

2 3.0S-Met group is excluded from regression analyses.

3 Significant differences (*P* < 0.05) between different Met sources (3.0S-OHMet vs. 3.0S-Met).

### 3.4 Met supplementation altered the amino acid metabolism of maternal and umbilical cord blood

As shown in Table 6, maternal serum arginine, lysine, leucine, isoleucine and valine concentrations increased linearly and quadratically (*P* < 0.05); serum hydroxyproline concentration increased linearly (*P* = 0.008); and serum phenylalanine and tyrosine concentrations increased quadratically (*P* < 0.05) following increased consumption of dietary OHMet at G114. Moreover, at G114, sows in the 3.0S-OHMet and 3.0S-Met groups showed significantly increased serum arginine, lysine, leucine, isoleucine, valine and threonine concentrations, and reduced glutamic acid concentration when compared to those in the CON and 1.5S-OHMet groups at G114 (*P* < 0.05). The consumption of a 1.5S-OHMet diet reduced phenylalanine concentration when compared to other diets (*P* = 0.001), and reduced tyrosine concentration when compared to levels following the consumption of 3.0S-OHMet- and 3.0S-Met-rich diets (*P* < 0.001).

**Table 6:** Effect of increased consumption of methionine on amino acid metabolism of sows at day 114 of gestation^1^.

Item	CON	1.5S-OHMet	3.0S-OHMet	3.0S-Met	SEM	*P*-value			
						ANOVA	Linear^2^	Quadratic^2^	Met source^3^
**EAA, μmol/L**									
Arginine	256.83^ab^	232.16^b^	295.48^a^	301.18^a^	19.194	0.042	0.017	0.010	0.818
Lysine	182.37^b^	142.22^b^	236.10^a^	275.20^a^	0.417	<0.001	0.058	0.006	0.179
Histidine	119.11	115.13	117.04	112.40	2.956	0.041	0.803	0.668	0.563
Tryptophan	60.51	69.51	59.10	59.08	6.061	0.551	0.597	0.105	0.551
Phenylalanine	174.31^a^	128.24^b^	205.70^a^	197.74^a^	13.836	0.001	0.323	0.002	0.960
Leucine	269.17^bc^	185.07^c^	337.76^ab^	404.14^a^	29.681	<0.001	0.012	0.004	0.174
Isoleucine	141.51^bc^	92.62^c^	191.64^ab^	231.26^a^	18.334	<0.001	0.005	0.003	0.132
Valine	294.40^b^	223.35^b^	435.92^ab^	507.48^a^	33.700	<0.001	0.017	0.005	0.349
Threonine	245.09^bc^	217.91^c^	311.98^ab^	350.88^a^	26.806	0.004	0.218	0.105	0.097
**NEAA, μmol/L**									
Glutamic acid	277.14^a^	244.49^a^	241.30^a^	158.92^b^	26.670	0.002	0.049	0.926	0.045
Glutamine	465.46	497.56	459.96	576.80	38.881	0.137	0.205	0.342	0.067
Aspartic acid	40.77	34.84	34.44	31.06	2.640	0.117	0.112	0.488	0.130
Carnosine	14.77	14.29	15.08	13.80	2.947	0.989	0.888	0.969	0.486
Alanine	777.94	829.36	704.56	696.89	49.563	0.185	0.315	0.150	0.908
Tyrosine	90.80^bc^	66.49^c^	118.14^b^	129.94^a^	9.267	<0.001	0.385	0.012	0.137
Hydroxyproline	72.25^a^	56.42^ab^	63.40^b^	45.96^b^	6.235	0.039	0.008	0.683	0.821
Proline	293.09	285.89	363.22	328.72	19.536	0.353	0.677	0.294	0.034

EAA = essential amino acid; NEAA = non-essential amino acid.

^a-c^ Within a row, means without a common superscript differ significantly (*P* < 0.05).

1 CON = basal diet; 1.5S-OHMet = basal diet + 1.5 g/kg OHMet; 3.0S-OHMet = basal diet + 3.0 g/kg OHMet; 3.0S-Met = basal diet + 3.0 g/kg Met. Each treatment contained 9 to 10 replicates (*n* = 9-10). Data were presented as means with standard error of mean (SEM).

2 3.0S-Met group is excluded from regression analyses.

3 Significant differences (*P* < 0.05) between different Met sources (3.0S-OHMet vs. 3.0S-Met).

As shown in Table 7, glutamine and aspartic acid concentrations in umbilical cord serum decreased linearly (*P* < 0.05) following the increased consumption of dietary OHMet. Sows fed 3.0S-OHMet diet had a higher hydroxyproline concentration and lower phenylalanine, leucine, Met, and ornithine concentrations than those fed the 3.0S-Met diet (*P* < 0.05).

**Table 7:** Effect of increased consumption of methionine on amino acid concentration of umbilical cord blood^1^.

Item	CON	1.5S-OHMet	3.0S-OHMet	3.0S-Met	SEM	*P*-value			
						ANOVA	Linear^2^	Quadratic^2^	Met source^3^
**EAA, μmol/L**									
Arginine	138.11	207.05	178.56	260.64	31.587	0.068	0.216	0.089	0.114
Lysine	322.26	299.25	268.09	273.07	27.878	0.516	0.216	0.912	0.891
Histidine	76.69	94.40	80.87	66.60	12.432	0.741	0.796	0.268	0.338
Tryptophan	9.46	3.50	2.93	3.07	1.948	0.118	0.053	0.336	0.916
Phenylalanine	127.03^b^	127.93^b^	120.10^b^	158.89^a^	9.600	0.028	0.524	0.635	0.019
Leucine	269.23	262.15	256.47	252.53	21.749	0.955	0.698	0.980	0.900
Isoleucine	78.11	77.13	82.12	119.75	8.186	0.240	0.747	0.780	0.012
Methionine	43.51	48.53	43.84	56.18	3.960	0.094	0.955	0.345	0.002
Valine	349.23	334.85	336.11	307.13	20.05	0.528	0.665	0.765	0.352
Threonine	225.09	202.75	211.93	174.98	17.04	0.235	0.636	0.512	0.149
**NEAA, μmol/L**									
Taurine	230.77	213.80	247.13	249.36	25.472	0.732	0.597	0.349	0.952
Serine	283.11	252.48	266.42	240.87	20.088	0.507	0.538	0.343	0.388
Glutamic acid	162.54	148.73	134.44	175.31	18.159	0.399	0.304	0.992	0.091
Glutamine	1093.54^a^	763.48^b^	780.64^b^	727.07^b^	73.223	0.007	0.008	0.075	0.540
Aspartic acid	52.40	35.50	34.33	45.62	5.071	0.060	0.031	0.254	0.083
Glycine	1294.06	1302.80	1236.20	1127.84	97.165	0.553	0.650	0.731	0.478
Alanine	1630.49	1485.80	1390.29	1207.00	101.451	0.143	0.141	0.857	0.473
Cysteine	47.66	46.83	37.65	36.71	4.758	0.148	0.105	0.403	0.828
Cystathionine	9.23	8.05	8.60	6.09	1.383	0.411	0.737	0.593	0.194
Tyrosine	82.09	69.95	61.16	80.93	7.955	0.094	0.077	0.865	0.059
Hydroxyproline	119.4^a^	96.03^ab^	94.89^b^	75.53^c^	9.320	0.026	0.111	0.391	0.036
Proline	331.31	262.70	251.40	274.87	25.627	0.178	0.066	0.429	0.477

EAA = essential amino acid; NEAA = non-essential amino acid.

^a-c^ Within a row, means without a common superscript differ significantly (*P* < 0.05). Data were presented as means with standard error of mean (SEM).

1 CON = basal diet; 1.5S-OHMet = basal diet + 1.5 g/kg OHMet; 3.0S-OHMet = basal diet + 3.0 g/kg OHMet; 3.0S-Met = basal diet + 3.0 g/kg Met. EAA = essential amino acid; NEAA = non-essential amino acid. Each treatment contained 9 to 10 replicates (*n* = 9-10).

2 3.0S-Met group is excluded from regression analyses.

3 Significant differences (*P* < 0.05) between different Met sources (3.0S-OHMet vs. 3.0S-Met).

### 3.5 Met supplementation increased the anti-oxidative capacity of maternal and umbilical cord blood

As shown in Table 8, following the increased consumption of dietary OHMet at G90, the serum the serum T-SOD activity increased linearly (*P* = 0.002), while the serum GSH-Px activity increased linearly (*P* = 0.001) and quadratically (*P* = 0.019). The MDA level in umbilical cord blood increased quadratically (*P* = 0.012), and the maternal GSH-Px activity (*P* = 0.030) increased linearly following increased consumption of dietary OHMet at G114. In addition, when compared to the 3.0S-Met group, 3.0S-OHMet supplementation increased the T-SOD (*P* = 0.046) and catalase (CAT) (*P* = 0.053) activities at G90 and the GSH-Px activity at G114 (*P* = 0.017).

**Table 8:** Effect of increased consumption of methionine on the oxidative status^1^.

Item	CON	1.5S-OHMet	3.0S-OHMet	3.0S-Met	SEM	*P*-value			
						ANOVA	Linear^2^	Quadratic^2^	Met source^3^
**G90**									
MDA, nmol/mL	2.21^ab^	2.74^a^	2.10^ab^	1.90^b^	0.207	0.045	0.751	0.047	0.342
T-SOD, U/mL	71.07^b^	71.56^b^	82.89^a^	75.32^b^	2.467	0.006	0.002	0.087	0.046
GSH-Px, U/mL	672.12^b^	656.06^b^	827.90^a^	798.62^a^	31.407	<0.001	0.001	0.019	0.547
CAT, U/mL	1.88	2.48	2.83	1.56	0.437	0.148	0.189	0.843	0.053
**G114**									
MDA, nmol/mL	4.36	3.67	3.94	4.03	0.313	0.531	0.368	0.229	0.848
T-SOD, U/mL	56.79	62.44	62.51	67.60	4.530	0.455	0.398	0.622	0.400
GSH-Px, U/mL	999.13	1113.40	1161.24	1034.86	44.175	0.070	0.030	0.580	0.017
CAT, U/mL	4.89	3.67	3.67	4.43	0.375	0.087	0.050	0.229	0.063
**Umbilical cord serum**									
MDA, nmol/mL	3.87	2.96	4.43	4.06	0.380	0.062	0.268	0.012	0.502
T-SOD, U/mL	42.06	51.11	46.99	52.14	4.037	0.331	0.323	0.137	0.384
GSH-Px, U/mL	117.58^b^	100.70^b^	135.14^ab^	169.94^a^	13.585	0.012	0.301	0.105	0.099
CAT, U/mL	2.92	1.8	2.97	2.39	0.430	0.239	0.940	0.080	0.392

G90 = day 90 of gestation; G114 = day 114 of gestation; MDA = malonaldehyde; T-SOD = total superoxide dismutase; GSH-Px = glutathione peroxidase; CAT = catalase.

^a,b^ Within a row, means without a common superscript differ significantly (*P* < 0.05). Data were presented as means with standard error of mean (SEM).

1 CON = basal diet; 1.5S-OHMet = basal diet + 1.5 g/kg OHMet; 3.0S-OHMet = basal diet + 3.0 g/kg OHMet; 3.0S-Met = basal diet + 3.0 g/kg Met. Each treatment contained 9 to 10 replicates (*n* = 9-10).

2 3.0S-Met group is excluded from regression analyses.

3 Significant differences (*P* < 0.05) between different Met sources (3.0S-OHMet vs. 3.0S-Met).

### 3.6 Multivariate analysis of the metabolite profiles

Metabolomics analysis was conducted to determine the alterations in maternal serum metabolic profiles at G114. The base peak chromatogram of a typical sample of sow serum showed significant differences (Fig. S2). The CON group had seven samples, and the 1.5S-OHMet, 3.0S-OHMet and 3.0S-Met groups had nine samples each group.

To further investigate the discrimination among the four treatment groups, a supervised orthogonal partial least squares-discriminant analysis (OPLS-DA) analysis was performed. The OPLS-DA score plots (Figs. S3A–C) showed the separation between the four groups by the first principal component in both positive and negative modes. The OPLS-DA fit criteria were calculated as follows: 1) CON vs. 1.5S-OHMet, *R*^2^X (cum) = 0.404, *R*^2^Y (cum) = 0.994, Q^2^ (cum) = 0.666 in the positive mode; *R*^2^X (cum) = 0.301, *R*^2^Y (cum) = 0.998, Q^2^ (cum) = 0.634 in the negative mode. 2) 1.5S-OHMet vs. 3.0S-OHMet, *R*^2^X (cum) = 0.362, *R*^2^Y (cum) = 0.981, Q^2^ (cum) = 0.869 in the positive mode; *R*^2^X (cum) = 0.288, *R*^2^Y (cum) = 0.985, Q^2^ (cum) = 0.747 in negative mode. 3) 1.5S-OHMet vs. 3.0S-Met, *R*^2^X (cum) = 0.377, *R*^2^Y (cum) = 0.992, Q^2^ (cum) = 0.946 in the positive mode; *R*^2^X (cum) = 0.380, *R*^2^Y (cum) = 0.996, Q^2^ (cum) = 0.923 in negative mode. Both *R*^2^Y and Q^2^ values were greater than 0.5, which indicated that the model was stable and reliable. The Q^2^ intercept values were less than 0.05, indicating that there was no overfitting.

### 3.7 Identification and functional annotation of the serum differential metabolites

**Fig. 1:**
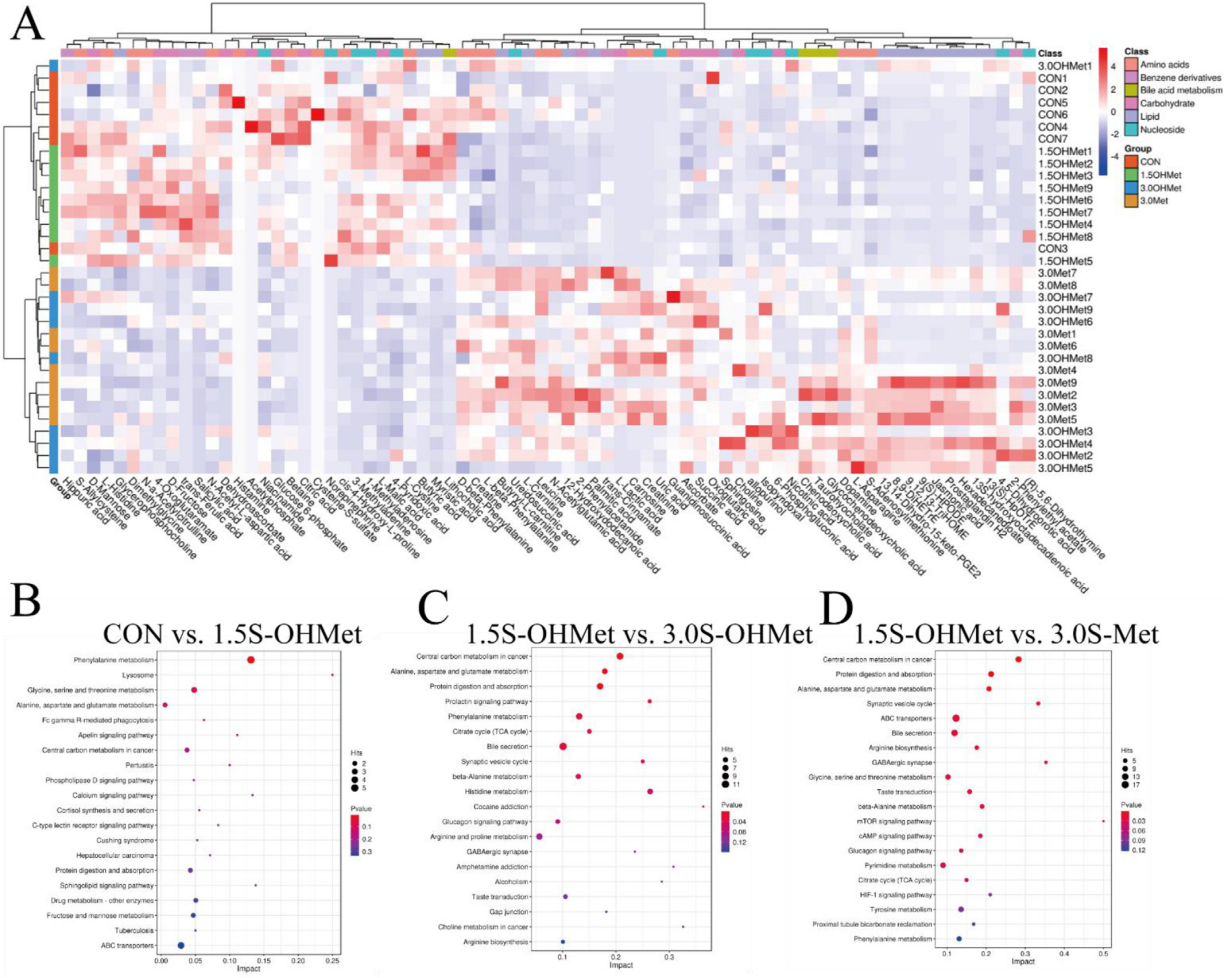
Effect of increased consumption of methionine as OHMet on serum metabolites of sows. (A) Distribution of differential serum metabolites in individual sows based on Pearson distance and average clustering (variable importance in projection [VIP] > 1.0, *P* < 0.05). (B), (C), (D) The KEGG pathway of differential metabolites between CON and 1.5S-OHMet, 1.5S-OHMet and 3.0S-OHMet, 1.5S-OHMet and 3.0S-Met. CON = basal diet; 1.5S-OHMet = basal diet + 1.5 g/kg OHMet; 3.0S-OHMet = basal diet + 3.0 g/kg OHMet; 3.0S-Met = basal diet + 3.0 g/kg Met.

We further screened the differential metabolites (DM) between the 1.5S-OHMet group and the other three groups based on the MS/MS analysis at G114 (Fig. S3 and Fig. S4, Table S1 and Table S2). The abundance of metabolites was normalized to those in the serum samples, and the threshold values of fold change ≥1.20 (or < 0.83) and a *P*-value <0.05 were used to determine the DM. A total of 75 DM were observed in a three-way comparison, and these were classified as amino acids (21 DM); benzene derivatives (6 DM); carbohydrates (14 DM); lipids (17 DM); bile acids, alcohols and derivatives (4 DM); and nucleotides (13 DM) (Fig. 1A). Further analysis revealed that dietary 1.5S-OHMet consumption elevated the serum contents of glucose-6-phosphate, citric acid, butyric acid, malic acid, 3-methyladenine, 1-methyladenosine, ferulic acid and salicylic acid, but reduced the serum contents of succinic acid, oxoglutaric acid, 9(S)-hydroperoxylinoleic acid, 13-hydroxy-octadecadienoic acid, uric acid and urea nitrogen when compared to contents observed in the 3.0S-OHMet and 3.0S-Met groups (*P* < 0.05, Fig. 1A and Table S1). Compared to the CON group, those fed 1.5S-OHMet group showed 18 upregulated and 52 downregulated DM (Fig. S3D and Table S3), and the most enriched pathways were “Phenylalanine metabolism”, “Lysosome” and “Alanine, aspartate and glutamate metabolism” (Fig. 1B and Table S4). Compared with the 3.0S-OHMet group, the 1.5S-OHMet group showed 73 upregulated and 127 downregulated DM (Fig. S3E and Table S3), and the most enriched pathways were “Central carbon metabolism in cancer”, “TCA cycle” and “Bile acid secretion” (Fig. 1C and Table S4). Compared with the 3.0S-Met-fed group, the 1.5S-OHMet group showed 81 upregulated and 151 downregulated DM (Fig. S3F and Table S3), and the most enriched pathways were “Central carbon metabolism in cancer”, “Bile acid secretion” and “TCA cycle” (Fig. 1D and Table S4).

These significantly different metabolites indicated the significantly different metabolic pathways reflected in the serum metabolome (Fig. 1B–D). The common metabolic pathways in the three-way comparison, were “Central carbon metabolism in cancer”, “Alanine, aspartate and glutamate metabolism”, “Protein digestion and absorption”, “Bile secretion”, “Citrate cycle (TCA cycle)” “Glycine, serine and threonine metabolism” and “Phenylalanine metabolism” (Table 9).

**Table 9:** The KEGG pathway of three comparison ways^1^.

Pathway network	Total	CON vs. 1.5S-OHMet		1.5S-OHMet vs. 3.0S-OHMet		1.5S-OHMet vs. 3.0S-Met	
		Hits	*P*-value	Hits	*P*-value	Hits	*P*-value
Central carbon metabolism in cancer	37	2	0.183	9	<0.001	11	<0.001
Alanine, aspartate and glutamate metabolism	28	1	0.117	6	0.006	7	0.002
Protein digestion and absorption	47	2	0.261	8	0.006	10	0.001
Bile secretion	97	–	–	11	0.030	13	0.016
Citrate cycle (TCA cycle)	20	1	0.348	4	0.029	4	0.047
Glycine, serine and threonine metabolism	50	3	0.087	–	–	8	0.021
Phenylalanine metabolism	60	5	0.008	8	0.026	7	0.123

Hits = the number of significant different metabolites in metabolic pathways.

1 CON = basal diet; 1.5S-OHMet = basal diet +1.5 g/kg OHMet; 3.0S-OHMet = basal diet + 3.0 g/kg OHMet; 3.0S-Met = basal diet +3.0 g/kg Met.

## 4 Discussion

To support the growth and development of their fetuses, mothers undergo significant physiological changes during pregnancy (Elango and Ball, 2016). Adequate levels of amino acids in pregnancy-related diets are essential for the health of mothers and their offspring. Ensuring sufficient amino acid intake is crucial for expectant mothers to safeguard their health of themselves and their progeny. The absorption and processing of Met is intricately associated with crucial processes such as methylation reactions, epigenetic regulation, redox balance, and fetal viability. However, excessive intake of Met through diets can indirectly affect fetal development by promoting Hcy accumulation. Given that OHMet shows enhanced utilization in parenteral tissues, undergoes facile metabolism through the trans-sulphuration, and reduces plasma Hcy content due to its distinct molecular structure (Xie et al., 2007; Fang et al., 2010b; Li et al., 2014), our present study aimed to investigate how increased consumption of Met in the form of OHMet enhances maternal metabolism and fetal survival during mid to late pregnancy.

**Fig. 2:**
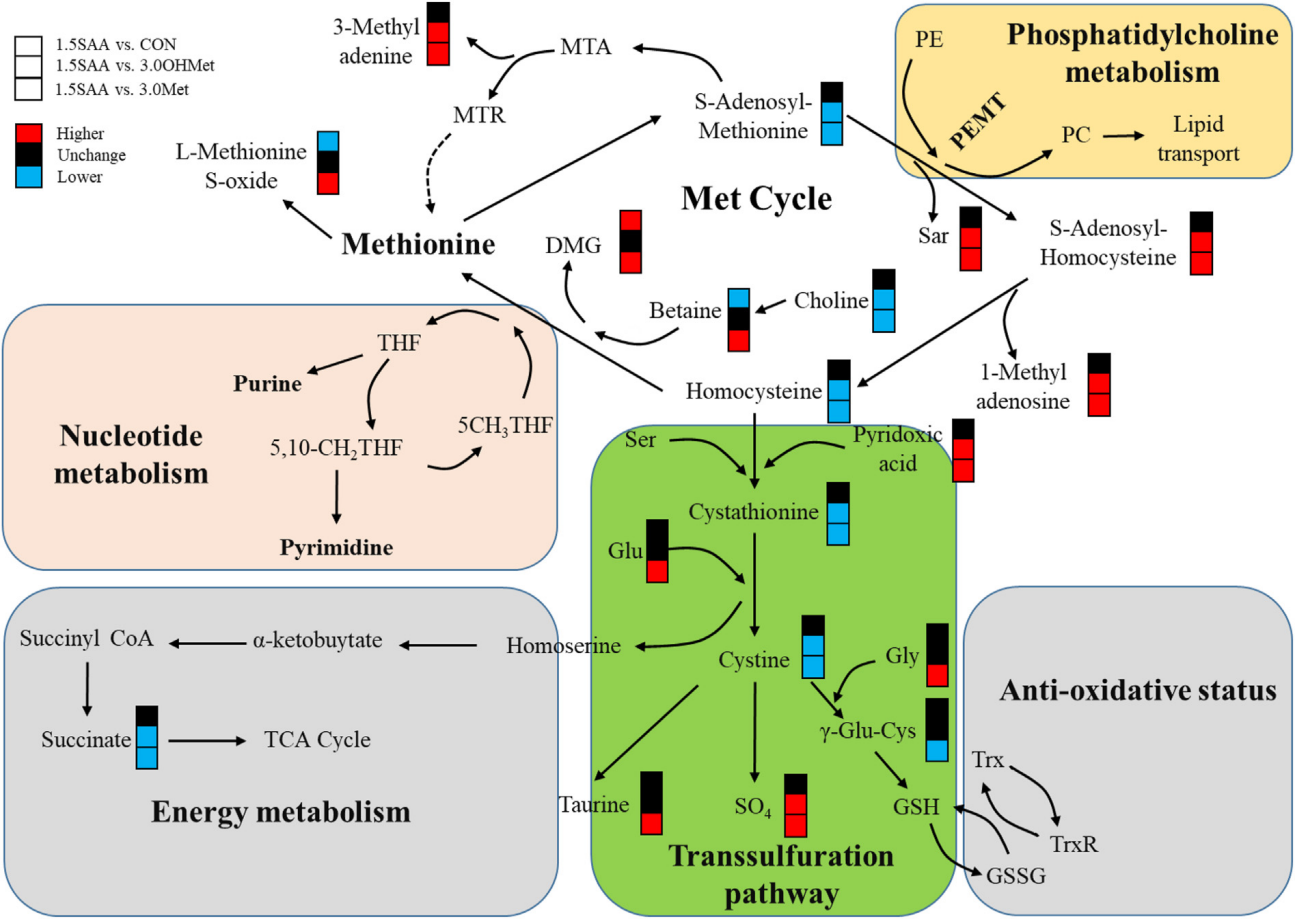
Different methionine metabolic pathways in maternal serum on day 114 of gestation. Red-colored symbols represent significant higher of metabolites in former group compared with the latter group, and black-colored symbols mean no difference, while blue-colored symbols indicate lower. CON = basal diet; 1.5S-OHMet = basal diet + 1.5 g/kg OHMet; 3.0S-OHMet = basal diet + 3.0 g/kg OHMet; 3.0S-Met = basal diet + 3.0 g/kg Met. DMG = dimethylglycine; MTA = methyl-thioadenosine; MTR = methionine synthase; THF = tetrahydrofolate; 5CH_3_THF = 5-methyltetrahydrofolate; 5,10-CH_2_THF = 5,10-methenyl-tetrahydrofolate; Sar = sarcosine; PE = phosphatidylethanolamine; PC = phosphatidylcholine; PEMT = phosphatidylethanolamine methyltransferase; Ser = serine; Glu = glutamate; γ-Glu-Cys = γ-glutamylcysteine; GSH = glutathione; GSSG = oxidized glutathione; Trx = thioredoxin; TrxR = thioredoxin reductase; SO_4_ = sulfate; TCA = tricarboxylic acid cycle.

An important finding in the present study was the elevation in piglet survival rates at farrowing with maternal supplementation of 1.5S-OHMet. This result was consistent with previous findings demonstrating enhanced fetal survival through dietary Met supplementation during late gestation (Bin et al., 2018; Xia et al., 2021). To further assess the effect of Met metabolism on reproductive performance following dietary supplementation with OHMet or Met during mid to late gestation, we analyzed the levels of maternal Met metabolites in sows fed with different Met-rich diets. Our findings revealed that maternal OHMet or Met consumption increased maternal Met and taurine concentration and decreased glycine concentrations at G90. A previous study revealed that Met was catabolized into cysteine and taurine, and glycine was required for this catabolic pathway (Rees et al., 2006b). To facilitate the conversion of Met to Hcy while maintaining an appropriate intracellular concentration of SAM, methyl groups are transferred from SAM to glycine (Blachier et al., 2020). Consistent with the present study, maternal Met supplementation was shown to enhance methylation processes through accelerated trans-methylation of Met. This observation was corroborated by the current study, which revealed that maternal consumption of 3.0S-OHMet and 3.0S-Met increased SAM concentrations and the SAM toSAH ratio in sows at G114. Previous research has indicated that a higher SAM to SAH ratio reflects a more favorable maternal one-carbon nutrient status, which is associated with greater cellular methylation potential in offspring (James et al., 2019; Korsmo and Jiang, 2021). However, we observed a significant increase in maternal Hcy concentration from mid to late gestation, which was consistent with a previous study (Xia et al., 2021). Notably, both the 3.0S-OHMet and 3.0S-Met groups exhibited significantly higher Hcy levels as compared to the CON and 1.5S-OHMet groups throughout the evaluation period. Moreover, a negative correlation was observed between Hcy levels and the survival rate of piglets. This observation may explain why maternal consumption of 3.0S-Met did not increase the fetal survival rate but instead elevated the stillborn ratio. Additionally, the concentrations of cystathionine, cysteine, and γ-glutamine-cysteine were increased, while those of pyridoxic acid and sulfate were reduced in the 3.0S-OHMet and 3.0S-Met groups at G114, thus indicating accelerated function of the trans-sulfuration pathway for clearing Hcy (Fig. 2). Intriguingly, maternal 3.0S-OHMet consumption showed lower Hcy concentration and higher cystathionine concentration compared with the 3.0S-Met group at G114, indicating that OHMet caused less accumulation of Hcy through the trans-sulfuration pathway compared to crystalline Met. These findings suggest that maternal supplementation with 3.0S-OHMet or 3.0S-Met promote the supply of one-carbon nutrients but result in increased Hcy production.

**Fig. 3:**
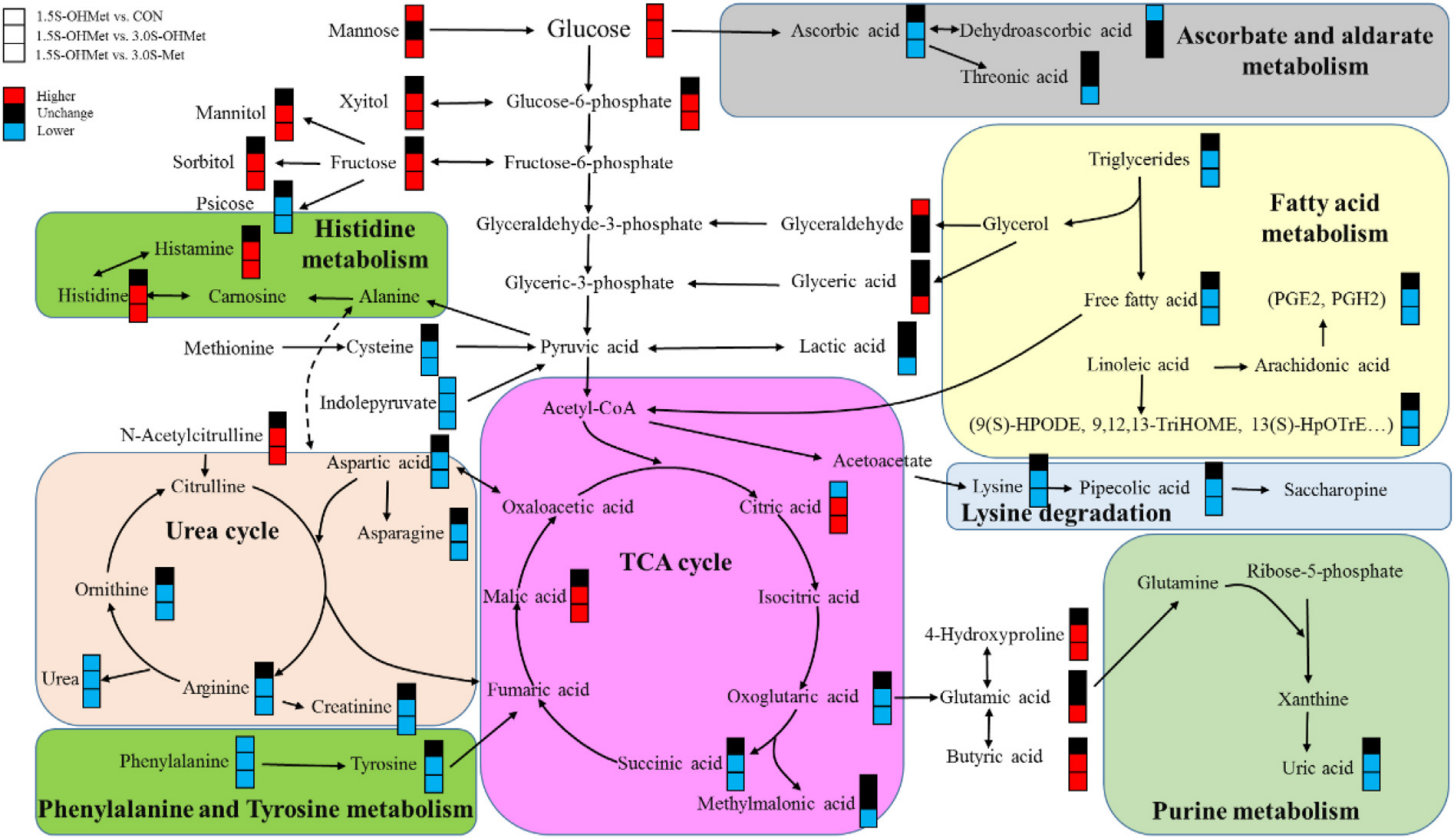
Different metabolic pathways in maternal serum on day 114 of gestation. Red-colored symbols represent significant higher of metabolites in former group compared with the latter group, and black-colored symbols mean no difference, while blue-colored symbols indicate lower. CON = basal diet; 1.5S-OHMet = basal diet + 1.5 g/kg OHMet; 3.0S-OHMet = basal diet + 3.0 g/kg OHMet; 3.0S-Met = basal diet + 3.0 g/kg Met. PGE2 = prostaglandin E2; PGH2 = prostaglandin H2; 9(S)-HPODE = hydroperoxylinoleic acid; 13(S)-HpOTrE = 13-hydroxy-octadecadienoic acid; 9,12,13-TriHOME = 9,12,13-trihydroxyoctadec-10-enoic acid; TCA = tricarboxylic acid cycle.

The Met metabolism intricately involves the Met cycle and the trans-sulfuration pathway, and exerts influence over various metabolic processes, including lipid metabolism, carbon metabolism, anti-oxidative activity, nucleotide metabolism, and amino acid metabolism (Fig. 3). Recent evidence underscores the regulatory role of Met metabolism in lipid metabolism, as it modulates the level of SAM, thereby influencing phosphatidylcholine (PC) synthesis through the activity of phosphatidylethanolamine methyltransferase (PEMT) (Watkins et al., 2003; Poloni et al., 2017; Blachier et al., 2020). Importantly, our study revealed significant alterations in sow lipid metabolites. Maternal supplementation with either 3.0S-OHMet or 3.0S-Met notably elevated the concentrations of TG, TC, LDL-C, and HDL-C in sows, along with TG concentrations in umbilical cord blood. Conversely, the 1.5S-OHMet group exhibited lower levels of hexadecanedioate, 8,9-DiHETrE, 9(S)-HPODE, 13(S)-HPOTrE, and carnitine, while displaying higher levels of butyric acid and myristic acid compared to the 3.0S-OHMet and 3.0S-Met groups (Fig. 3). Notably, 13(S)-HPOTrE serves as a primary precursor to 4-hydroxynonenal, a lipid peroxidation product, that undergoes further metabolism to 9(S)-HPODE (Ayala et al., 2014). These findings imply that sows fed-1.5S-OHMet diet had enhanced antioxidative capacity compared to those in the 3.0S-OHMet and 3.0S-Met groups. In addition, given that butyric acid, a short-chain fatty acids, facilitates energy supply and fetal delivery, and carnitine plays an indispensable role in transporting long-chain fatty acids (Wei et al., 2019), it can be inferred that maternal lipid mobilization was intensified in the 3.0S-OHMet and 3.0S-Met groups as compared to that in the 1.5S-OHMet group.

Previous research has demonstrated that an elevated concentration of Hcy increases the expression of cholesterol 7a-hydroxylase via peroxisome proliferator-activated receptor α, thereby affecting bile acid (BA) metabolism (Marrapodi and Chiang, 2000). Notably, intrahepatic cholestasis during pregnancy is a significant factor that contributes to fetal mortality (McIlvride et al., 2017). In the present study, metabolome analysis revealed alterations in maternal bile acid secretion following supplementation with 3.0S-OHMet or 3.0S-Met. Furthermore, a positive correlation was observed between TBA and Hcy concentrations (Fig. S1D), which suggested that a potential link between increased Hcy levels and maternal TBA production. Subsequent analysis indicated higher levels of glycochenodeoxycholic acid (GCDCA) and chenodeoxycholic acid, along with lower levels of lithocholic acid (LCA), in the 3.0S-OHMet and 3.0S-Met groups. A noteworthy observation is that gut microbes related to bile acid metabolism can facilitate the conversion of GCDCA to LCA (Honda et al., 2020). These results suggested that excessive Met supplementation might affect gut microbial composition, thus interfering with fetal survival through changes in BA metabolism. This partly explains the higher fetal survival rate in the 1.5S-OHMet group than in the 3.0S-OHMet and 3.0S-Met groups. Further research is warranted to determine the effect of Met metabolism on microbial metabolism during late gestation.

Dietary supplementation with 1.5S-OHMet significantly increased the maternal glucose concentration during farrowing. Methionine, a glycogenic amino acid, undergoes metabolism to succinyl-CoA through the trans-sulfuration pathway (Martínez et al., 2017), which is then converted into glucose through gluconeogenesis (Hicks et al., 2023). The tricarboxylic acid (TCA) cycle is regarded as the most efficient mechanism for animals to produce energy, and a byproduct of this cycle is nicotinamide-adenine dinucleotide phosphate (NADP) (He et al., 2019). Nicotinic acid serves as a substrate for the synthesis of nicotinamide-adenine dinucleotide (NAD) and NADP (Ivers et al., 1993). Elevated nicotinic acid levels in 1.5S-OHMet sows suggested an enhancement of the TCA cycle to generate more energy, which is consistent with the results of a previous study (Bin et al., 2018). Metabolomics analysis revealed that sows fed with 3.0S-OHMet or 3.0S-Met had lower concentrations of glucose-6-phosphate, citric acid, and malic acid but higher levels of succinic acid and oxoglutaric acid compared to those fed 1.5S-OHMet (Fig. 3). These results indicate that maternal 1.5S-OHMet consumption elevates the levels of energy metabolites and facilitates increased energy supply during fetal delivery by promoting the TCA cycle. Additionally, our previous study demonstrated a higher plasma glucose level in pigs fed the OHMet diet than in those fed the Met group (Zhang et al., 2015). The 1.5S-OHMet-fed sows exhibited lower levels of lactic acid than those fed 3.0S-Met. During farrowing, the uterus induces the release of a significant amount of lactic acid into the bloodstream (Feyera et al., 2018). In this regard, the elevated butyric acid level may aid in the clearance of lactic acid by accelerating blood flow (Fang et al., 2009), thereby promoting fetal survival. These results indicate that maternal 1.5S-OHMet consumption increases glucose levels and provides more energy. However, excessive supplementation of Met may cause an upsurge in succinate production, thereby potentially disrupting energy metabolism (Chen et al., 2020).

Pregnancy is a process of oxidative stress, and the degree of oxidative stress intensifies in the late gestation (Berchieri-Ronchi et al., 2011). The metabolism of Met is closely associated with antioxidant capacity. It can activate the mechanistic target of rapamycin complex 1 (mTORC1) signaling pathway by SAM or produce GSH and Taurine through the trans-sulfuration pathway (Zhang et al., 2018; Rasch et al., 2020; Zhong et al., 2021). In the current study, maternal serum T-SOD and GSH-Px activities increased with dietary Met supplementation at G90, thus indicating that Met consumption improved oxidative status. These findings aligns with our previous study, which demonstrated that Met consumption protected intestinal development against oxidative stress (Zhong et al., 2016). In addition, the elevated cysteine concentration enhanced maternal antioxidant capacity (Li et al., 2014; Ding et al., 2019). However, the elevated Hcy concentrations may have compromised antioxidant capacity in the 3.0S-OHMet and 3.0S-Met groups. Intriguingly, compared to the 3.0S-Met group, maternal 3.0S-OHMet consumption was associated with higher T-SOD and CAT activities at G90, and with higher GSH-Px activity at G114. Our results suggested that Hcy levels were negatively correlated with the average weight of piglets born alive. These results also indicate that sows in the 3.0S-OHMet group possessed stronger anti-oxidative capacity than those in the 3.0S-Met group, which was one of the important factors that contributed to the reduced rate of low birth weight.

The fourth important finding demonstrated an enhanced nucleotide metabolism following 1.5S-OHMet supplementation. SAM serves as a vital source of one-carbon units, which were closely associated with fetal nucleotide synthesis (Gao et al., 2019; Korsmo and Jiang, 2021). Nucleotides are crucial components of nucleic acids, and an imbalance in their levels can contribute to various diseases (Lane and Fan, 2015). Uric acid, produced during purine metabolism by xanthine oxidase, has a profound effect on oxidative stress, inflammation, and enzymes associated with glucose and lipid metabolism (Glantzounis et al., 2005). The administration of allopurinol, a xanthine oxidoreductase inhibitor, can effectively reduce serum uric acid levels and provide protective effects in oxidative stress conditions (Glantzounis et al., 2005). The higher levels of uric acid and allopurinol observed in sows fed 3.0S-OHMet or 3.0S-Met indicated a compensatory increase in allopurinol due to elevated uric acid levels. These findings suggested that 1.5S-OHMet-fed sows had lower oxidative stress levels than those fed-3.0S-OHMet or fed-3.0S-Met. Furthermore, a decrease in serum levels of 3-methyladenine and 1-methyladenosine was observed in the 3.0S-OHMet-fed or 3.0S-Met-fed sows as compared to that in the 1.5S-OHMet-fed sows. 3-Methyladenine functions by inhibiting phosphatidylinositol 3-kinase, blocking autophagy synthesis in lysozymes (Slavin et al., 2018; Huang et al., 2022). The increased levels of 1-methyladenosine in the 1.5S-OHMet group may have promoted placental translation of methylated mRNA (Wang et al., 2021). Taken together, maternal consumption of 1.5S-OHMet contributes to maternal health improvement by enhancing nucleotide metabolism.

As late gestation progresses, the amino acid requirement of sows undergoes a significant increase, due to rapid fetal development (Maynard et al., 2021). Changes in the metabolism of Met can affect the metabolism of other amino acids. Another intriguing finding is that maternal Met supplementation altered protein digestion and absorption. In addition, a positive correlation between serum levels of leucine, isoleucine, arginine, valine, lysine, phenylalanine, tyrosine, threonine, and methionine metabolites was observed (Fig. S5C). Accumulating evidence suggests that SAM, arginine and leucine can activate the mTORC1 signaling pathway by inhibiting SAMTOR, CASTOR1, and sestrin2, respectively (Saxton et al., 2016; Gu et al., 2017; Cangelosi et al., 2022). The mTORC1 signaling pathway not only directed placental metabolism and growth but also influenced the development of offspring (Akhaphong et al., 2021). The elevated levels of SAM, arginine, and leucine may activate the placental mTORC1 signaling pathway, thereby promote fetal development. Arginine is recognized as a key controller of placental angiogenesis, and maternal supplementation with arginine enhanced the placental synthesis of nitric oxide and polyamine, increased angiogenesis, and augmented water and amino acid transport to improve conceptus development and survival (Herring et al., 2022). We also found that 1.5S-OHMet consumption increased the maternal levels of salicylic acid, ferulic acid, mannose, and fructose, and decreased the phenylalanine level as compared to those in the CON, 3.0S-OHMet and 3.0S-Met groups (Fig. 3). A previous study has provided evidence that phenylalanine was metabolized to salicylic acid and ferulic acid (Ennis et al., 2020). Ferulic acid can mitigate oxidative damage (Montaser et al., 2019). The lower levels of phenylalanine, along with the higher levels of salicylic acid and ferulic acid in the 1.5S-OHMet-fed sows, suggested enhanced phenylalanine catabolism and increased antioxidant capacity. Additionally, the supplementation of maternal Met significantly influenced the metabolism of histidine and creatinine during the mid to late gestation period. At G114, both 3.0S-OHMet and 3.0S-Met groups exhibited lower levels of histamine and higher levels of carnosine than the 1.5S-OHMet-fed sows (Fig. 3). Histamine serves as a crucial precursor to carnosine in muscle tissues and specific regions of the brain, where carnosine plays a pivotal role as a buffer and antioxidant (Brosnan and Brosnan, 2020). This disparity suggests that the sows fed with either 3.0S-OHMet or 3.0S-Met might have utilized a greater amount of carnosine to combat oxidative stress compared to the 1.5S-OHMet-fed sows. Creatinine, which serves as an indicator of muscle catabolism (Yang et al., 2009), exhibited significantly higher levels in both 3.0S-OHMet and 3.0S-Met groups compared with the 1.5S-OHMet group. Previous research has already postulated that an increase in creatinine concentration is directly associated with the mobilization of stored proteins and, indirectly, with the fat level in the body mass (Van Niekerk et al., 1963). Taken together, these results suggest that the consumption of 1.5S-OHMet improves maternal amino acid metabolism.

## 5 Conclusion

The current study provided new insights into the mechanism wherein sows fed a 1.5S-OHMet diet during mid to late gestation showed an elevated fetal survival rate. This improvement was attributed to a combination of elevated maternal levels of glucose, ferulic acid, salicylic acid, glucose-6-phosphate, citric acid, butyric acid, malic acid, 3-methyladenine, and 1-methyladenosine, with reduced levels of succinic acid, oxoglutaric acid, 9(S)-HPODE, 13(S)-HPOTrE, uric acid and urea nitrogen. In contrast, the elevated Hcy level may lead to the decreased fetal survival rate after maternal consumption with 3.0S-OHMet or 3.0S-Met, despite an increase in the SAM levels and the SAM toSAH ratio. These findings have important implications for enhancing maternal and fetal health through the optimal maternal consumption of Met from appropriate sources during mid to late gestation.

## CRediT authorship contribution statement

**Rui Zhou:** Visualization, Writing – original draft. **Li Zhe:** Investigation, Visualization. **Yves Mercier:** Investigation, Visualization. **Liang Hu:** Investigation, Methodology. **Ran Li:** Investigation, Methodology. **Hong Chen:** Investigation, Methodology. **Xiaoling Zhang:** Conceptualization, Methodology. **Lingjie Huang:** Conceptualization, Methodology. **Lun Hua:** Supervision, Writing – review & editing. **Yong Zhuo:** Supervision, Writing – review & editing. **Jian Li:** Supervision, Writing – review & editing. **Shengyu Xu:** Supervision, Writing – review & editing. **Yan Lin:** Supervision, Writing – review & editing. **Bin Feng:** Supervision, Writing – review & editing. **Lianqiang Che:** Supervision, Writing – review & editing. **De Wu:** Supervision, Writing – review & editing. **Zhengfeng Fang:** Funding acquisition, Writing – review & editing.

## Declaration of competing interest

We declare that we have no financial and personal relationships with other people or organizations that can inappropriately influence our work, and there is no professional or other personal interest of any nature or kind in any product, service and/or company that could be construed as influencing the content of this paper.
