# Supplementary material for: Serum metabolomics analysis reveals a novel association between maternal metabolism and fetal survival in sows fed diets containing differing methionine levels and sources
Source: Anim Nutr. 2024 Oct 26;20:145–57. doi: 10.1016/j.aninu.2024.07.008 (PMC11833788; doi:10.1016/j.aninu.2024.07.008)
Supplement: Multimedia component 1 [file mmc1.docx]

**Table S1** Identification of differential serum metabolites from sows of CON, 3.0S-OHMet and 3.0S-Met compared with 1.5S-OHMet sows.

| Item | CON vs. 1.5S-OHMet | | | 1.5S-OHMet vs. 3.0S-OHMet | | | 1.5S-OHMet vs. 3.0S-Met | | | Class |
| --- | --- | --- | --- | --- | --- | --- | --- | --- | --- | --- |
|  | log2FC^1^ | *P*^2^ | VIP^3^ | log2FC^1^ | *P*^2^ | VIP^3^ | log2FC^1^ | *P*^2^ | VIP^3^ |  |
| cis-4-Hydroxy-L-proline | - | - | - | 1.72 | <0.001 | 1.62 | 1.72 | <0.001 | 1.55 | Amino acids |
| N-a-Acetylcitrulline | - | - | - | 1.08 | 0.024 | 1.09 | 1.26 | 0.019 | 1.10 | Amino acids |
| N-Acetyl-L-aspartic acid | - | - | - | 0.90 | 0.008 | 1.55 | 1.77 | <0.001 | 1.53 | Amino acids |
| N-Acetylglutamic acid | 1.61 | 0.003 | 1.96 | -1.86 | <0.001 | 1.67 | -3.20 | <0.001 | 1.79 | Amino acids |
| Leucine | - | - | - | -2.23 | 0.001 | 1.47 | -2.40 | <0.001 | 1.54 | Amino acids |
| L-Asparagine | - | - | - | -2.41 | 0.040 | 1.25 | -1.91 | 0.002 | 1.29 | Amino acids |
| Guanidinosuccinic acid | - | - | - | -2.92 | 0.004 | 1.63 | -2.08 | 0.001 | 1.44 | Amino acids |
| Betaine | 1.66 | 0.002 | 1.95 | - | - | - | 1.62 | 0.019 | 1.05 | Amino acids |
| Dimethylglycine | -1.13 | 0.008 | 1.67 | - | - | - | 1.90 | 0.002 | 1.40 | Amino acids |
| Cysteine-S-sulfate | 6.71 | 0.023 | 1.73 | 1.79 | 0.004 | 1.66 | - | - | - | Amino acids |
| S-Allylcysteine | - | - | - | 1.47 | 0.006 | 1.64 | 1.52 | 0.002 | 1.38 | Amino acids |
| S-Adenosylmethionine | - | - | - | -0.69 | 0.002 | 1.53 | -0.69 | <0.001 | 1.47 | Amino acids |
| L-Cystine | - | - | - | 0.92 | 0.008 | 1.36 | 1.18 | <0.001 | 1.58 | Amino acids |
| L-Histidine | - | - | - | 0.99 | 0.003 | 1.43 | 1.42 | <0.001 | 1.70 | Amino acids |
| Histamine | - | - | - | 3.16 | <0.001 | 1.70 | 3.09 | <0.001 | 1.56 | Amino acids |
| Carnosine | - | - | - | -4.23 | <0.001 | 1.79 | -5.51 | <0.001 | 1.82 | Amino acids |
| Creatine | - | - | - | -1.19 | 0.006 | 1.48 | -1.40 | <0.001 | 1.43 | Amino acids |
| Creatinine | - | - | - | -5.49 | <0.001 | 2.04 | -5.61 | <0.001 | 1.66 | Amino acids |
| L-beta-phenylalanine | 0.40 | 0.003 | 1.87 | -3.14 | 0.003 | 1.61 | - | - | - | Amino acids |
| D-beta-phenylalanine | - | - | - | - | - | - | -0.95 | <0.001 | 1.37 | Amino acids |
| Dopamine | - | - | - | -1.69 | 0.003 | 1.47 | -1.87 | 0.003 | 1.38 | Amino acids |
| 2-Phenylacetamide | - | - | - | -1.82 | <0.001 | 1.76 | -2.90 | <0.001 | 1.78 | Benzene derivatives |
| trans-Cinnamate | - | - | - | -1.97 | 0.014 | 1.61 | -2.48 | 0.001 | 1.39 | Benzene derivatives |
| 2-Phenylethyl acetate | 2.92 | 0.002 | 2.17 | -4.07 | <0.001 | 1.68 | -4.77 | <0.001 | 1.83 | Benzene derivatives |
| trans-Ferulic acid | -1.62 | 0.031 | 1.38 | 3.22 | <0.001 | 1.64 | 3.34 | <0.001 | 1.60 | Benzene derivatives |
| Salicylic acid | -0.72 | 0.008 | 1.67 | 2.01 | <0.001 | 2.06 | 3.22 | <0.001 | 1.92 | Benzene derivatives |
| Hippuric acid | - | - | - | 0.89 | 0.004 | 1.64 | 1.11 | <0.001 | 1.48 | Benzene derivatives |
| Dehydroascorbate | 1.05 | 0.042 | 1.41 | - | - | - | - | - | - | Carbohydrate |
| D-Mannose | -0.47 | 0.042 | 1.49 | - | - | - | 0.48 | <0.001 | 1.45 | Carbohydrate |
| 4-Oxoglutaramate | - | - | - | 3.06 | <0.001 | 1.85 | 3.45 | <0.001 | 1.82 | Carbohydrate |
| Citric acid | 2.66 | 0.001 | 2.21 | 1.80 | 0.003 | 1.84 | 3.00 | <0.001 | 1.72 | Carbohydrate |
| D-Fructose | - | - | - | 1.38 | 0.008 | 1.24 | 2.75 | <0.001 | 1.64 | Carbohydrate |
| L-Malic acid | - | - | - | 0.98 | 0.003 | 1.81 | 1.32 | <0.001 | 1.78 | Carbohydrate |
| Glucose 6-phosphate | - | - | - | 0.66 | 0.024 | 1.36 | 1.18 | 0.001 | 1.43 | Carbohydrate |
| Ascorbate | - | - | - | -0.76 | 0.004 | 1.55 | -0.71 | 0.001 | 1.49 | Carbohydrate |
| 6-Phosphogluconic acid | - | - | - | -1.09 | 0.014 | 1.29 | -1.02 | 0.008 | 1.15 | Carbohydrate |
| Succinic acid | - | - | - | -1.38 | 0.003 | 1.38 | -0.97 | 0.004 | 1.13 | Carbohydrate |
| Oxoglutaric acid | - | - | - | -2.30 | <0.001 | 1.63 | -1.60 | 0.002 | 1.35 | Carbohydrate |
| Choline | - | - | - | -3.07 | <0.001 | 1.67 | -2.95 | 0.001 | 1.33 | Carbohydrate |
| L-Lactic acid | - | - | - | - | - | - | -6.10 | 0.001 | 1.69 | Carbohydrate |
| Acetylphosphate | - | - | - | 5.33 | 0.004 | 1.34 | 5.66 | 0.003 | 1.33 | Carbohydrate |
| Butyric acid | - | - | - | 3.08 | <0.001 | 1.63 | 2.53 | 0.002 | 1.44 | Lipid |
| Myristic acid | - | - | - | 1.14 | <0.001 | 1.83 | 1.35 | <0.001 | 1.79 | Lipid |
| Glycerophosphocholine | - | - | - | 0.77 | 0.014 | 1.29 | 1.08 | 0.003 | 1.37 | Lipid |
| Sphingosine | - | - | - | -0.71 | 0.040 | 1.15 | -0.59 | 0.040 | 1.11 | Lipid |
| 13S-hydroxyoctadecadienoic acid | - | - | - | -1.29 | 0.002 | 1.55 | -1.78 | <0.001 | 1.49 | Lipid |
| L-Carnitine | - | - | - | -1.30 | 0.003 | 1.65 | -1.78 | <0.001 | 1.85 | Lipid |
| 12-Hydroxydodecanoic acid | 1.23 | <0.001 | 2.49 | -2.01 | <0.001 | 2.19 | -2.80 | <0.001 | 1.62 | Lipid |
| 9(S)-HPODE | - | - | - | -2.19 | 0.014 | 1.48 | -3.13 | <0.001 | 1.52 | Lipid |
| 9,12,13-TriHOME | - | - | - | -2.19 | 0.011 | 1.49 | -3.14 | <0.001 | 1.53 | Lipid |
| 13,14-Dihydro-15-keto-PGE2 | - | - | - | -2.30 | 0.008 | 1.44 | -2.65 | <0.001 | 1.49 | Lipid |
| Prostaglandin H2 | - | - | - | -2.64 | 0.002 | 1.44 | -3.36 | 0.001 | 1.46 | Lipid |
| 13(S)-HpOTrE | - | - | - | -2.69 | 0.024 | 1.20 | -2.53 | 0.006 | 1.31 | Lipid |
| 8,9-DiHETrE | - | - | - | -2.80 | 0.002 | 1.46 | -3.73 | 0.001 | 1.53 | Lipid |
| Hexadecanedioate | - | - | - | -3.58 | 0.002 | 1.67 | -4.30 | 0.001 | 1.43 | Lipid |
| Palmitic acid | - | - | - | - | - | - | -1.67 | <0.001 | 1.55 | Lipid |
| Butyryl-L-carnitine | - | - | - | -4.01 | 0.011 | 1.30 | -5.14 | <0.001 | 1.79 | Lipid |
| Jasmonic acid | 3.48 | 0.001 | 1.94 | -6.34 | <0.001 | 1.79 | -7.20 | <0.001 | 1.73 | Lipid |
| Lithocholic acid | - | - | - | 1.84 | 0.001 | 1.63 | 1.64 | 0.001 | 1.54 | Bile acid metabolism |
| Glycochenodeoxycholic acid | - | - | - | -1.44 | 0.024 | 1.21 | -2.68 | 0.004 | 1.34 | Bile acid metabolism |
| Chenodeoxycholic acid | - | - | - | -2.78 | 0.003 | 1.67 | -3.24 | <0.001 | 1.50 | Bile acid metabolism |
| Taurohyocholate | - | - | - | - | - | - | -2.43 | 0.040 | 1.02 | Bile acid metabolism |
| Niacinamide | - | - | - | - | - | - | 0.55 | 0.011 | 1.17 | Nucleoside |
| Nicotinic acid | -1.14 | 0.042 | 1.54 | - | - | - | - | - | - | Nucleoside |
| 3-Methyladenine | - | - | - | 2.23 | <0.001 | 1.97 | 2.26 | <0.001 | 1.87 | Nucleoside |
| 1-Methyladenosine | - | - | - | 0.59 | 0.001 | 1.54 | 0.68 | <0.001 | 1.48 | Nucleoside |
| 4-Pyridoxic acid | - | - | - | 0.55 | 0.014 | 1.59 | 0.92 | <0.001 | 1.80 | Nucleoside |
| Pyridoxamine | - | - | - | 0.24 | 0.020 | 1.23 | 0.23 | 0.006 | 1.21 | Nucleoside |
| 4,5-Dihydroorotic acid | - | - | - | -0.53 | 0.004 | 1.36 | -0.18 | 0.024 | 1.11 | Nucleoside |
| (R)-5,6-Dihydrothymine | - | - | - | -1.49 | 0.004 | 1.56 | -1.35 | 0.011 | 1.29 | Nucleoside |
| Isopyridoxal | - | - | - | -1.59 | 0.003 | 1.45 | -0.84 | 0.024 | 1.16 | Nucleoside |
| allopurinol | - | - | - | -2.52 | 0.001 | 1.46 | -2.20 | 0.006 | 1.34 | Nucleoside |
| Ureidosuccinic acid | 1.96 | 0.023 | 1.74 | -2.93 | <0.001 | 1.70 | -3.71 | <0.001 | 1.86 | Nucleoside |
| Uric acid | - | - | - | -3.37 | 0.006 | 1.54 | -3.77 | <0.001 | 1.75 | Nucleoside |
| Norepinephrine | - | - | - | 3.78 | <0.001 | 1.82 | 4.41 | <0.001 | 1.73 | Nucleoside |

CON = basal diet; 1.5S-OHMet = basal diet + 1.5 g/kg OHMet; 3.0S-OHMet = basal diet + 3.0 g/kg OHMet; 3.0S-Met = basal diet + 3.0 g/kg Met.

^1^ Fold change (FC) values were obtained from mean peak area of former sow/mean peak area of latter sow. If the log2FC value is greater than 0, it means that metabolite level is higher in former group compared with the latter group.

^2^*P*-values were calculated from student's *t*-test with a threshold of 0.05.

^3^ Variable importance in the projection (VIP) values were obtained from Partial Least Squares-Discriminant Analysis (PLS-DA) models with a threshold of 1.


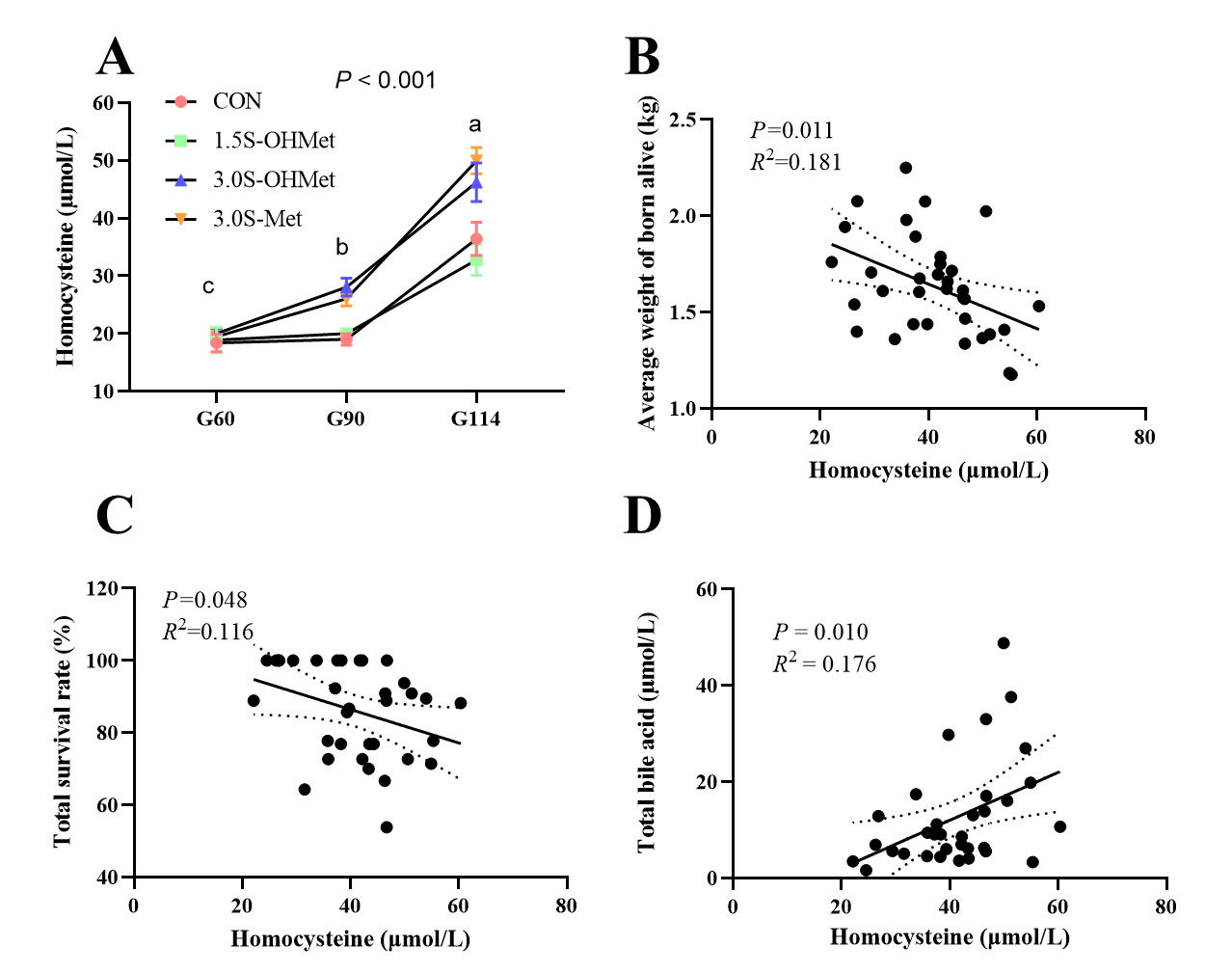


Fig. S1 (A) The changed trend in homocysteine (Hcy) in serum along with the gestation process. (B) The correlation analysis between Hcy content and average weight of born alive at G114. (C) The correlation analysis between Hcy content and total survival rate of piglets at G114. (D) The correlation analysis between Hcy and total bile acid (TBA) contents at G114. CON = basal diet; 1.5S-OHMet = basal diet + 1.5 g/kg OHMet; 3.0S-OHMet = basal diet + 3.0 g/kg OHMet; 3.0S-Met = basal diet + 3.0 g/kg Met. G60 = day 60 of gestation; G90 = day 90 of gestation; G114 = day 114 of gestation. Data are presented as the means ± SEM. ^a-c^ Means with different letters differ significantly (*P* ＜ 0.05).


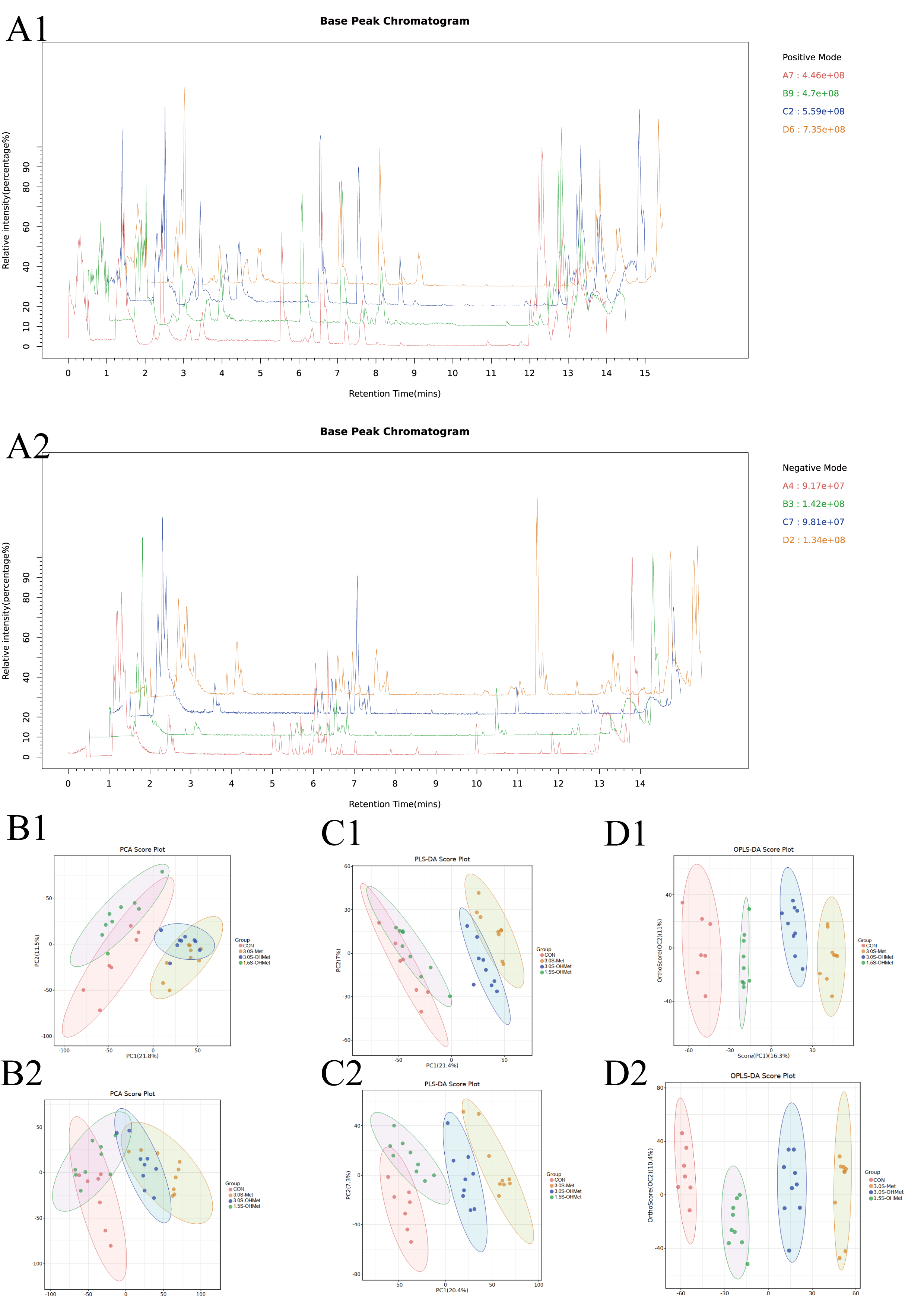


Fig. S2 The base peak chromatogram of a typical sample of sow serum (A) and metabolome score plots for Principal Component Analysis (PCA) (B), Partial Least Squares-Discriminant Analysis (PLS-DA) (C) and Orthogonal Partial Least Squares-Discriminant Analysis (OPLS-DA) (D). Panels (A1, B1, C1, D1) are ESI+, Panels (A2, B2, C2, D2) are ESI−, respectively. CON = control group (*n* = 7); 1.5S-OHMet = 1.5 g/kg OHMet (*n* = 9); 3.0S-OHMet = 3.0 g/kg OHMet (*n* = 9); 3.0S-Met (3.0S) = 3.0 g/kg Met (*n* = 9);


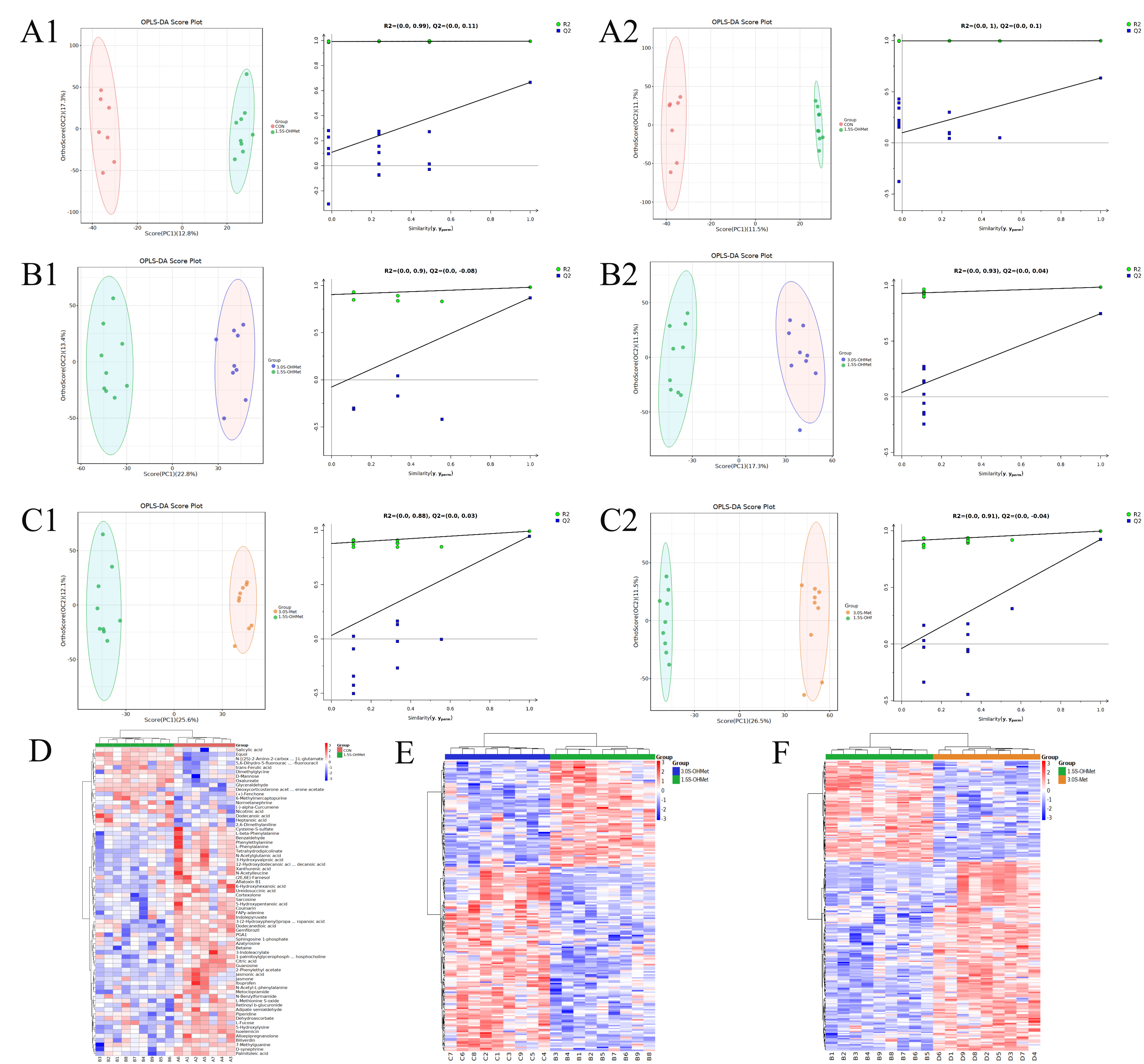


Fig. S3 Orthogonal Partial Least Squares-Discriminant Analysis (OPLS-DA) loading plots and clustering heat map based on the serum metabolic profiles. (A), (B), (C) The OPLS-DA plot and OPLS-DA replacement inspection chart between CON and 1.5S-OHMet, 1.5S-OHMet and 3.0S-OHMet, 1.5S-OHMet and 3.0S-Met. Panels (A1, B1, C1) are ESI+, panels (A2, B2, C2) are ESI−, respectively). CON = basal diet; 1.5S-OHMet = basal diet + 1.5 g/kg OHMet; 3.0S-OHMet = basal diet + 3.0 g/kg OHMet; 3.0S-Met = basal diet + 3.0 g/kg Met.


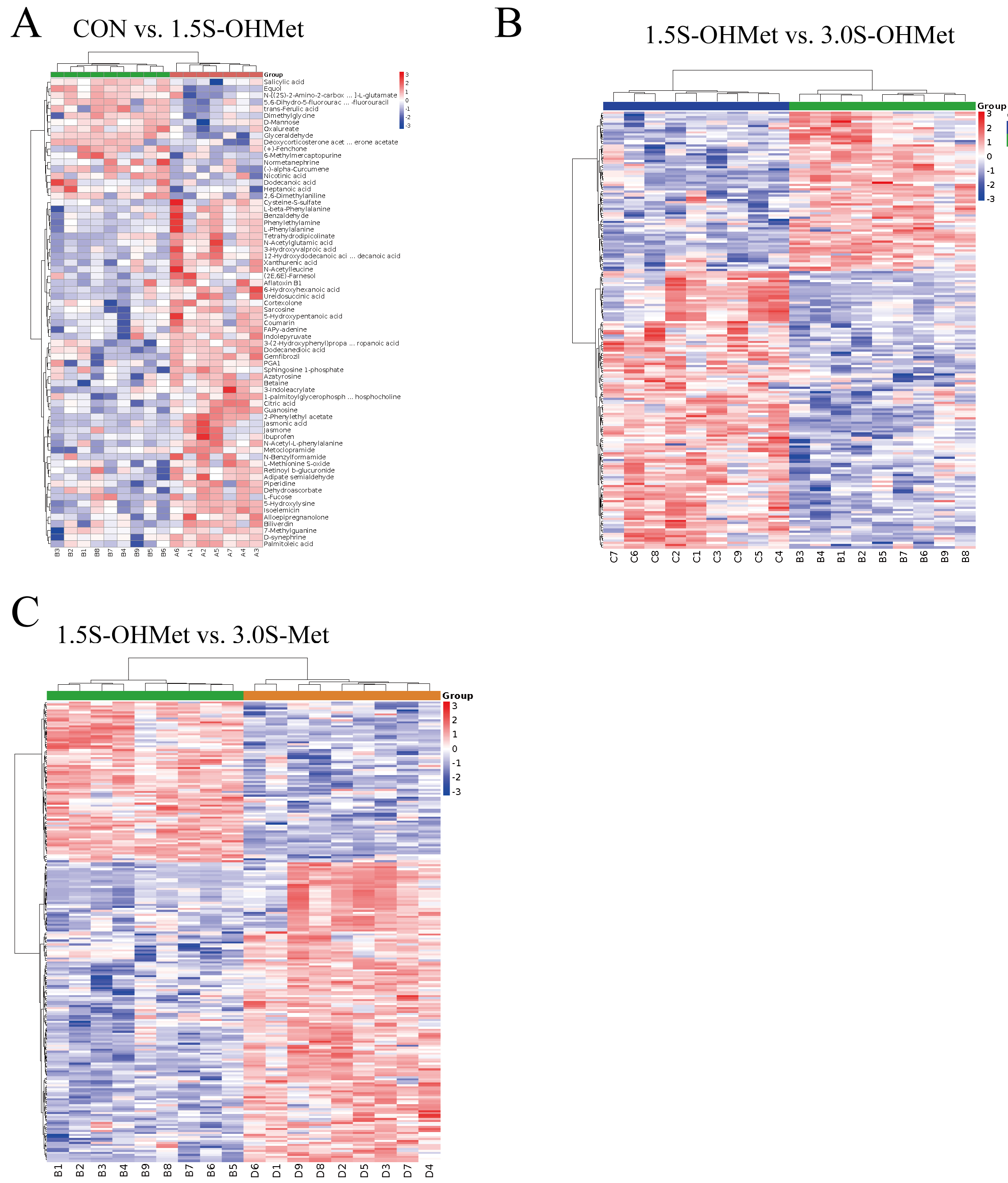


Fig. S4 The clustering heat map based on the serum metabolic profiles. (A), (B), (C) Clustering hotmap of the different metabolites in the serum between CON and 1.5S-OHMet, 1.5S-OHMet and 3.0S-OHMet, 1.5S-OHMet and 3.0S-Met. CON = basal diet; 1.5S-OHMet = basal diet + 1.5 g/kg OHMet; 3.0S-OHMet = basal diet + 3.0 g/kg OHMet; 3.0S-Met = basal diet + 3.0 g/kg Me


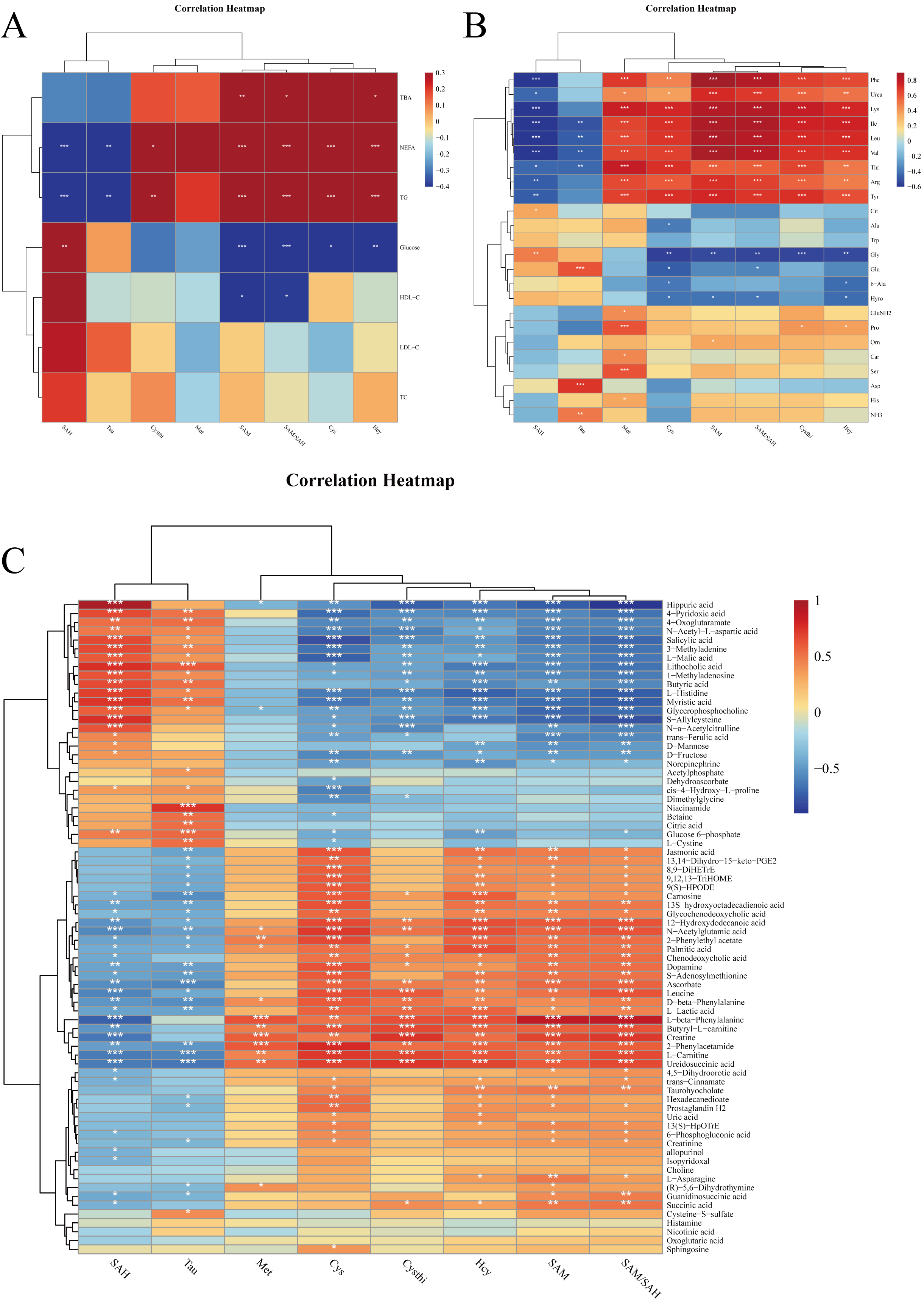


Fig. S5 (A) The correlation between methionine metabolites and glycolipid metabolites of sows at G114. (B) The correlation between methionine metabolites and amino acid concentration of sows at G114. (C) The correlation between methionine metabolites and serum metabolites of sows at G114. CON = basal diet; 1.5S-OHMet = basal diet + 1.5 g/kg OHMet; 3.0S-OHMet = basal diet + 3.0 g/kg OHMet; 3.0S-Met = basal diet + 3.0 g/kg Met. SUN = serum urea nitrogen; GLU = glucose; TG = triglyceride; NEFA = non-esterified fatty acids; TC = total cholesterol; LDL-C = low density lipoprotein cholesterol; HDL-C = high density lipoprotein cholesterol, TBA = total bile acid; SAM = S-adenosyl-methionine; SAH = S-adenosyl-homocysteine; Hcy = homocysteine; Met = methionine; Cys = cysteine; Cysyhi = cystathionine; Tau = taurine; Ser = serine; Gly = glycine; Arg = arginine; Lys = lysine; His = histidine; Try = tryptophan; Phe = phenylalanine; Leu = leucine; Ile = Isoleucine; Val = valine; Thr = Threonine. **P* < 0.05, ***P* < 0.01, *** *P* < 0.001.
